# Identification and Affinity Determination of Protein-Antibody and Protein-Aptamer Epitopes by Biosensor-Mass Spectrometry Combination †

**DOI:** 10.3390/ijms222312832

**Published:** 2021-11-27

**Authors:** Loredana-Mirela Lupu, Pascal Wiegand, Daria Holdschick, Delia Mihoc, Stefan Maeser, Stephan Rawer, Friedemann Völklein, Ebrahim Malek, Frederik Barka, Sascha Knauer, Christina Uth, Julia Hennermann, Wolfgang Kleinekofort, Andreas Hahn, Günes Barka, Michael Przybylski

**Affiliations:** 1 Centre for Analytical Biochemistry and Biomedical Mass Spectrometry (AffyMSLifeChem), and Steinbeis Transfer Centre for Biopolymer Analysis and Biomedical Mass Spectrometry, Marktstrasse 29, 65428 Rüsselsheim am Main, Germany; loredanalupu92@gmail.com (L.-M.L.); pascal.wiegand@stw.de (P.W.); daria.holdschick@student.hs-rm.de (D.H.); mihoc555@gmail.com (D.M.); st.maeser@gmx.de (S.M.); st.rawer@gmx.de (S.R.); eeby@gmx.net (E.M.); wolfgang.kleinekofort@hs-rm.de (W.K.); 2 Department of Engineering & Institute for Microtechnologies (IMTECH), RheinMain University, 65428 Rüsselsheim am Main, Germany; friedemann.voelklein@hs-rm.de; 3 Sunchrom GmbH, Industriestr. 18, 61381 Friedrichsdorf, Germany; fbarka@sunchrom.de (F.B.); gbarka@sunchrom.de (G.B.); 4 Sulfotools GmbH, Bahnhofsplatz 1, 65428 Rüsselsheim am Main, Germany; knauer@sulfotools.com (S.K.); uth@sulfotools.com (C.U.); 5 Department of Pediatrics, Universitätsmedizin Mainz, 55130 Mainz, Germany; julia.hennermann@unimedizin-mainz.de; 6 Department of Child Neurology, Justus-Liebig-University Giessen, Feulgenstraße 10-12, 35389 Giessen, Germany; andreas.hahn@paediat.med.uni-giessen.de

**Keywords:** monoclonal, polyclonal protein antibodies, DNA aptamers, epitope structure determination, proteolytic epitope extraction, affinity determination, SPR, chip-MALDI-mass spectrometry, myoglobin, interleukin-8, cross-immunoreactivity

## Abstract

Analytical methods for molecular characterization of diagnostic or therapeutic targets have recently gained high interest. This review summarizes the combination of mass spectrometry and surface plasmon resonance (SPR) biosensor analysis for identification and affinity determination of protein interactions with antibodies and DNA-aptamers. The binding constant (K_D_) of a protein–antibody complex is first determined by immobilizing an antibody or DNA-aptamer on an SPR chip. A proteolytic peptide mixture is then applied to the chip, and following removal of unbound material by washing, the epitope(s) peptide(s) are eluted and identified by MALDI-MS. The SPR-MS combination was applied to a wide range of affinity pairs. Distinct epitope peptides were identified for the cardiac biomarker myoglobin (MG) both from monoclonal and polyclonal antibodies, and binding constants determined for equine and human MG provided molecular assessment of cross immunoreactivities. Mass spectrometric epitope identifications were obtained for linear, as well as for assembled (“conformational”) antibody epitopes, e.g., for the polypeptide chemokine Interleukin-8. Immobilization using protein G substantially improved surface fixation and antibody stabilities for epitope identification and affinity determination. Moreover, epitopes were successfully determined for polyclonal antibodies from biological material, such as from patient antisera upon enzyme replacement therapy of lysosomal diseases. The SPR-MS combination was also successfully applied to identify linear and assembled epitopes for DNA–aptamer interaction complexes of the tumor diagnostic protein C-Met. In summary, the SPR-MS combination has been established as a powerful molecular tool for identification of protein interaction epitopes.

## 1. Introduction

In recent years, antibodies have found wide-spread application for diagnostics as well as therapy, and currently constitute the fastest growing group of medication with sales volumes up to USD 100 billion (in 2018) [1,2,3,4]. Antibodies also contribute a dominant share to disease diagnostics, being almost indispensable in laboratory assays, such as ELISA, Western Blot, and biosensor analysis [4,5,6]. Biosensor techniques using antibodies, such as surface plasmon resonance (SPR) are well established for detection and quantification of biomolecular interactions. Although mass spectrometry (MS) is an established tool for molecular characterization of biomolecular interactions [6,7], the combination of biosensor technologies and mass spectrometry is still in an initial state of development and application [6,7,8,9]. Recently, biosensor and mass spectrometry technologies are gaining increasing interest as “hybrid” tools for detection, chemical structure determination and quantification of protein–antibody interactions, particularly recognition epitopes of antibodies [4,6,7,9]. 

The molecular characterization of antibody epitopes of a peptide or protein antigen is a key element for the discovery of biomarkers, vaccines and in drug development [4,6,7,10,11,12]. It is well established that antibodies bind to different peptide or protein ligands via linear (sequence) or assembled (“conformational”) epitopes [4]. Several approaches have been developed in recent years and are currently explored for epitope identification. Classical methods, such as X-ray crystallography and NMR spectroscopy, require relatively large amounts of material with high purity [13,14]. On the other hand, mass spectrometry (MS) based methods, together with proteolytic digestion, have been developed using hydrogen–deuterium exchange of antigen–antibody complexes (HDX), chemical cross-linking, fast photochemical oxidation of proteins (FPOP), and alanine scanning of proteolytic peptide fragments [15,16,17,18,19,20,21,22,23,24,25]. More recently, the combination of biosensor analysis and proteolytic affinity-mass spectrometry has been developed and explored for the identification of antibody epitopes. The major approach of this technology, proteolytic excision, and/or extraction [26] utilizes the specific digestion of a protein from an immune complex, generation of specific peptide fragments of a protein antigen and their presentation to an immobilized antibody, in order to isolate the epitope peptides that bind to the antibody and their identification by mass spectrometry (PROTEX-MS) (Figures S1 and S2). The feasibility and efficiency of the PROTEX-MS approach has been already demonstrated in a number of bioanalytical applications, and in biomedical studies of mono- and polyclonal antibody-protein complexes [27,28,29,30,31,32,33,34].

Biosensor technologies such as surface plasmon resonance (SPR) and surface-acoustic wave (SAW) have been successfully employed for quantitative analyses of affinity-bound ligands [35,36,37]. However, a principal limitation of biosensors is their lack of providing chemical structure information [8,9]. Mass spectrometry alone does not provide quantitative and/or kinetic affinity determinations. For the identification and affinity determination of biomolecular interaction epitopes, efficient methods have been developed using the PROTEX-MS combination. This review will focus on the proteolytic epitope extraction-MS approach that provides the direct affinity determination from an SPR chip and epitope identification using MALDI-MS. In recent studies, mass spectrometric identifications of epitopes have been reported for a number of affinity pairs, such as protein- antibody [29,30,31,32], oligosaccharides-lectin [33,34], and most recently, aptamer-protein complexes.

## 2. Results and Discussion 

### 2.1. SPR-MALDI-MS Combination for Epitope and Affinity Determination

**Figure 1:**
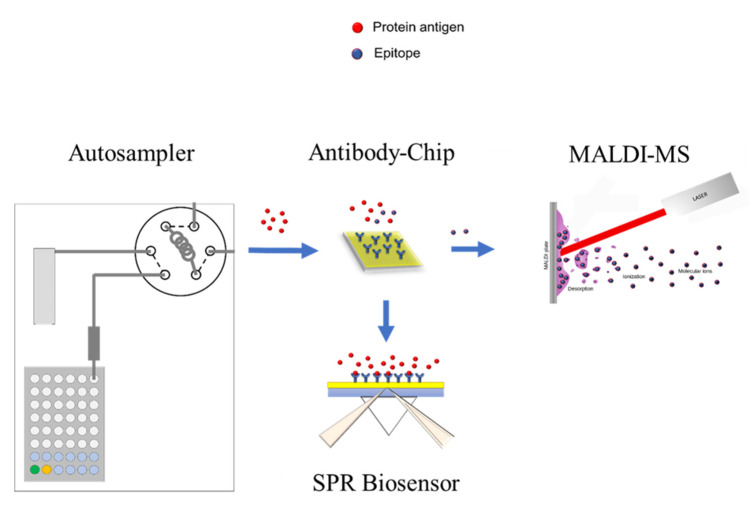
Scheme of the analytical platform for the Chip-SPR–MALDI-MS epitope analyzer. Autosampler unit with SPR chip—MALDI-MS connection and transfer line from SPR valve to MALDI-MS sample target (multiwell plate). Sample injection is performed on the SPR chip containing the immobilized antibody. Peptides injected on the antibody channel provide the SPR sensorgram of the binding epitope(s), and eluted epitope peptide(s) are spotted on the MALDI target plate. K_D_ determinations of the antibody-bound protein and peptide fragments are performed by injecting a dilution series over the SPR chip.

**Figure 2:**
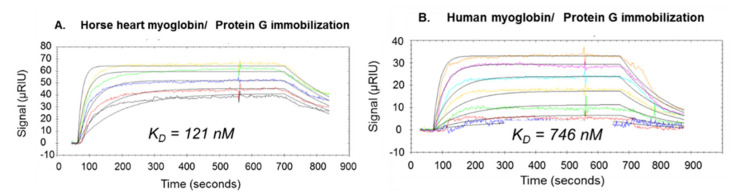
SPR interaction kinetics of horse heart myoglobin (**A**) and human myoglobin (**B**) with polyclonal anti-MG antibody immobilized on protein G [38].

Figure 1 shows a scheme of the analytical platform of the SPR chip–MALDI-MS combination, comprising an autosampler unit connected to an SPR chip, connection of autosampler and SPR biosensor, and a transfer line from the SPR to a MALDI-MS sample target. The SPR-MS combination can utilize all major biosensor types and microfluidic systems suited for combination with mass spectrometry, such as prism-based SPR and SAW-biosensors. An SPR aqueous fluidic module compatible with both ESI-MS and MALDI-MS has been developed and optimized for submission of a protein or peptide digestion mixture from the autosampler onto the SPR chip containing the immobilized antibody. The SPR sensorgram is determined in a first step, and K_D_ determination of the chip-immobilized antibody interacting with the epitope peptides is performed by injecting a dilution series of peptides at different concentrations over the SPR chip (s. examples in Figure 2). Following removal of unbound components by washing, the epitope(s) are eluted at slightly acidic aqueous conditions and spotted on the MALDI target plate. 

The proteolytic epitope extraction-MS approach has been successfully applied to a number of epitope identifications of monoclonal, as well as polyclonal antibodies [6,7,38]. Two antibody immobilization methods were explored: (i), random immobilization on activated surface-assembled monolayer (SAM) chips; and (ii), oriented antibody immobilization using the F_c_ binding of protein G [38] (Figure S3). The comparison of both methods clearly showed that immobilization using covalent crosslinking at the Fc region to protein G was superior to standard immobilization with regard to long-term antibody stabilities; reproducibility; antibody consumption for immobilization; and non-specific binding to the surface [32,38] (Figure S3).

In bioanalytical applications, immobilization using protein G from preactivated Sepharose bead columns, and from SPR chips was highly suitable to provide epitope and affinity determinations with high sensitivity [38] (Figure 2; Table 1). Using EDC/NHS activation on the SPR chip, protein G coupling can be performed in volatile aqueous buffer (ammonium-acetate or -bicarbonate) and provides stable crosslinking of the antibody to the Fc region. Injection of ethanolamine as blocking buffer was used to obtain stable antibody surfaces. Antibody SPR chips prepared in this manner showed remarkable stabilities over more than 3 weeks. Ligand immobilization on CNBr-activated Sepharose columns employed a similar procedure for different affinity pairs (myoglobin antibodies; DNA-aptamers), as described in the subsequent chapters.

Control SPR studies were carried out with the intact protein antigens to ensure stable immobilization, and control tests with blank columns ascertained the absence of unspecific interactions. Using the proteolytic extraction procedure for epitope analysis, incubation, washing, and elution of the intact proteins was directly analyzed by MALDI-MS. 

### 2.2. Determination of Monoclonal and Polyclonal Antibody Epitopes of Myoglobin

**Figure 3:**
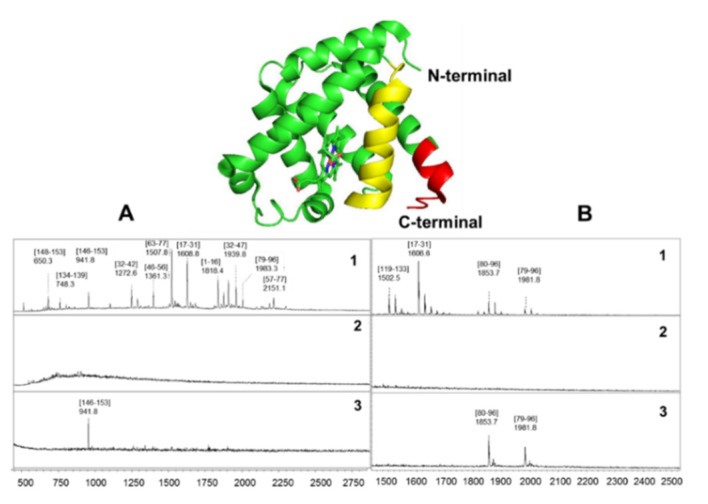
Identification of epitopes of horse heart myoglobin by chip-SPR-MALDI-MS with a monoclonal antibody (**A**) and a polyclonal antibody (**B**). Antibodies were immobilized by protein G- covalent fixation as described in Materials and Methods, and tryptic digest mixtures of myoglobin submitted on the chip. Sequence of MALDI spectra for each antibody denote the supernatant (1a), background after final washing (2), and elution with 0.01 M aqueous TFA (3). Epitope peptides identified are illustrated in the myoglobin structure, (146–153) for monoclonal (red) [38], and (79–96) for the polyclonal antibody (yellow).

The oxygen binding Myoglobin (MG) is one of the major proteins expressed in skeletal and cardiac muscle as a single cytoplasmic heme protein of 153 amino acids [38,39,40,41] (Figure S4). Due to its functional similarity to hemoglobin, MG reversibly binds oxygen and facilitates oxygen transport from red blood cells to mitochondria, with a single O_2_-binding site and a hyperbolic saturation curve. The three-dimensional structure of MG has been described in detail [38], with 75% of the main chain being folded in an α-helical conformation (Figure 3). MG has recently gained increased interest due to its potential role as a biomarker for acute myocardial infarct (AMI), in comparison to corresponding alternative biomarkers [41]. Following cardiac muscle injury, both cardiac and skeletal serum levels of MG are substantially increased and are also elevated in certain disorders by release of myoglobin from damaged or necrotic muscle cells [38]. Acute Myocardial Infarct (AMI) affects millions of people each year, and rapid, precise, and timely diagnosis is considered of key importance for early therapeutic intervention [40]. The application of monoclonal MG antibodies has been a major tool in immunoassays for MG determination in serum or plasma of AMI patients. Immunoassays have been employed for diagnosis and quantification of myocardial necrosis and infarction [38,39]. High immunological cross reactivity has been described for several MG analogues, such as horse heart and human MG. 

For the identification and affinity determination of biomolecular interaction epitopes of MG, efficient methods have been developed and employed by combination of proteolytic extraction using PROTEX-MS. Both monoclonal and polyclonal anti-MG antibodies were immobilized on self-assembled monolayer (SAM)-SPR affinity chips, as well as on Sepharose affinity microcolumns, and incubated with proteolytic digestion mixtures of horse heart and human MG, respectively (Figure S5). After washing off non-binding peptides, the fractions eluted by mild acidification were analyzed by MALDI-MS and revealed the presence of a single linear epitope peptide, respectively. SPR determinations of the MG–antibody complex with the intact myoglobin(s) revealed high affinities for a polyclonal anti-horse MG antibody with binding constants K_D_ of approximately 120 and 740 nM for horse heart and human MG, respectively (Figure 2; Table 1) [38]. For a monoclonal anti-horse heart MG antibody, the comparison of cross reactivities showed considerably lower affinity of horse heart as compared to human MG. 

Epitope identifications of horse heart MG to a monoclonal and a polyclonal antibody by proteolytic epitope extraction-MS using the SPR–MS combination are shown in Figure 3A,B. For both antibodies, identical results were obtained by using two complementary modes, respectively; (i), with the Sepharose-immobilized antibody bound to an affinity microcolumn, and (ii), binding of atryptic digestion mixture of MG to the SPR chip containing the immobilized antibody. In both analyses, single linear epitopes were identified. A µg aliquot of the tryptic digestion mixture was incubated with each antibody, and unbound peptides were washed away. The corresponding supernatant peptides were analyzed by MALDI-MS, and the final washing fractions did not show residual peptides (Figure 3; trace 1, 3). Elution from the SPR chip with 0.1% TFA (pH 3) provided a single peptide, respectively, that was identified as the C-terminal epitope sequence (146–153) for the monoclonal antibody (YKELGFQG), while the epitope sequence (79–96) (KGHHEAELKPLAQSHAT K) was identified for the polyclonal antibody. The epitope peptides found in excess in the supernatant fractions are readily explained by the non-stoichiometric ratios of the MG digest–to antibody complexes, containing typically a molar excess of the protein component in the digestion mixture [7,26,27]. These epitope determinations clearly demonstrate the efficiency of the SPR–MS combination for the molecular affinity and epitope characterization from both mono- and polyclonal antibodies, suggesting the affinity-MS approach as a powerful tool for identifying epitope-based diagnostic biomarkers.

### 2.3. Protein G Immobilization for Antibody Epitope Determination from Biological Material

Immobilization of antibodies using directed, covalent attachment with protein G on preactivated Sepharose columns and SPR chips has been recently explored as a highly efficient tool in epitope and affinity studies from biological material, such as serum and plasma antibodies [7,8,42]. Bioanalytical applications were carried out in studies of pathophysiological antibodies upon protein replacement therapy in lysosomal diseases, and of anti-drug antibodies against therapeutic antibody drugs [43,44]. Rheumatoid arthritis (RA) is an autoimmune disease that causes inflammation and eventual destruction of joints [44,45]. TNF-alpha blocking protein therapeutics have been successfully used in RA therapy, such as the monoclonal antibody, Adalimumab (ADA) [44,45,46]. Adalimumab, as well as related human anti-murine antibodies, however, cause high immunogenicity associated with the formation of antibodies, that can decrease therapeutic effects, neutralize the drug antibody, and frequently require immunosuppressive intervention. Thus, the identification of ADA antibody epitopes should be useful to neutralize the antibody effects and develop improved diagnostics. 

**Figure 4:**
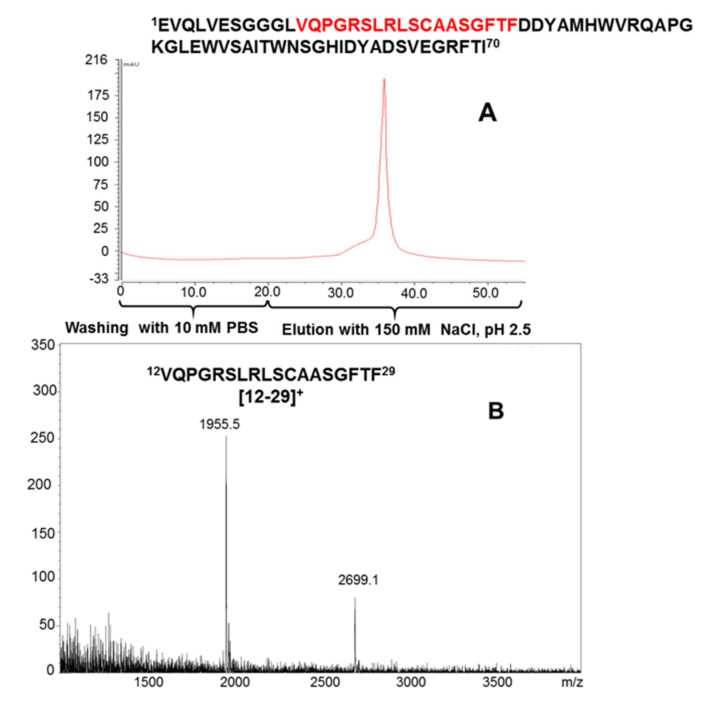
Elution of α-chymotryptic digest mixture of Adalimumab from immobilized serum (**A**) and MALDI-MS identification of the epitope peptide (12–29) (**B**).

Proteolytic epitope extraction in combination with MALDI-MS was applied to identify the epitopes recognized by ADA antibodies. Antibody immobilization of the IgG enriched serum by DMP crosslinking was performed with a POROS protein G sensor cartridge, using the identical procedure as employed for the immobilization of myoglobin antibodies [38,41,45,46,47,48,49]. Separation of ADA on a serum IgG-affinity column showed a homogeneous peak at 280 nm eluted at 32.2 min (Figure 4A), indicating binding of ADA to the immobilized serum antibodies. For epitope identification, proteolytic extraction was employed with a chymotryptic digest mixture submitted to the affinity column, and non-binding peptide fragments were removed by washing (supernatant fraction). Elution by mild acidification (pH 3) and MALDI-MS provided a single peptide corresponding to the ADA heavy chain sequence (12–29) [49]. The epitope was ascertained by additional fragmentation and MS analysis, and was located at the begin of the CDR1 region (Figure 4B and Figure S6).

### 2.4. Identification of Assembled (Discontinuous) Antibody Epitopes of Interleukin-8

The 72-amino acid polypeptide chemokine Interleukin-8 (IL8; CXCL8) plays an important role in immunological and inflammatory processes in humans and mammalian species. IL8 has been shown to be involved in rheumatoid arthritis and several chronic inflammatory diseases [50,51,52]. The interaction of IL8 with its natural receptors, CXCR1 and CXCR2 plays a crucial role in these diseases, as shown in recent studies [52]. A murine monoclonal anti-human IL8 antibody has been shown to inhibit the IL8–CXCR1 interaction; however, the binding epitope of the interaction has been hitherto unknown. 

**Figure 5:**
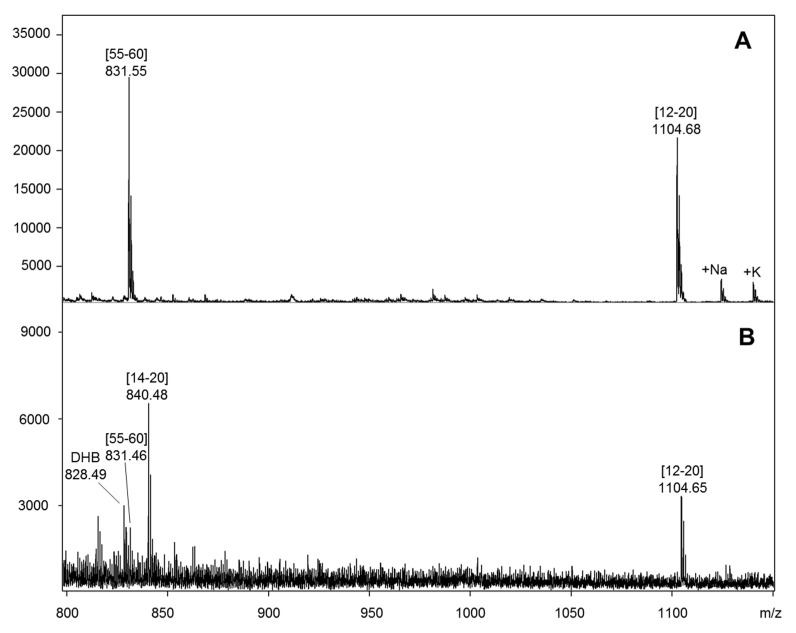
Identification of the interleukin-8 epitope to a monoclonal anti-IL8 antibody by proteolytic extraction MALDI-MS, upon elution of the bound epitopes from a microaffinity column (**A**), and elution from an SPR chip (**B**). The antibody was immobilized by NHS/EDC coupling as described [53]. Epitope elution provided a discontinuous epitope comprising IL-8 sequences (12–20) and (55–60).

For the identification of antibody epitope(s), proteolytic epitope extraction was applied (i), with the antibody immobilized on a Sepharose micro-affinity column, and (ii), by immobilization on an SPR chip (Figure 1). Following removal of excess supernatant [38] at pH 7, no unbound peptides were found in the last washing fraction. Subsequent elution performed at slightly acidified solution and MALDI-MS analysis of the elution fractions from the IL8 tryptic digestion mixture revealed two distinct epitope peptides, IL8 (12–20) (TYSKPFHPK) and IL8 (55–60) (ENWVQR) [53]. These epitope sequences ascertained an assembled (discontinuous) epitope structure comprising two spatially adjacent IL8 sequences (Figure 5). Since previous studies had shown that the anti-IL8 antibody is inhibitory to the IL8-CXCR1 interaction, the overlapping interactions are consistent with the inhibiting effect of the antibody to the IL8-CXCR1 interaction (Figures S7 and S8).

Although a direct determination of binding constants of each separate epitope was not possible, an SPR analysis by injection of the elution fraction containing the epitope peptides over the immobilized anti-IL8 antibody chip indicated specific binding of the epitopes to the antibody. Further confirmation of the epitopes was obtained by SPR determination of the synthetic peptides with the immobilized anti-IL8 antibody, which revealed affinities within a K_D_ range of 5 to 20 µM. 

### 2.5. Epitope Determination of Pathophysiological Antibodies upon Enzyme Replacement Therapy of Lysosomal Storage Diseases

Lysosomal storage diseases (LSDs) constitute a group of approximately 60 enzymes, most of them carrying out specific hydrolysis reactions in lysosomes [54,55,56,57,58,59,60,61]. Mutations in the gene and/or biochemical changes lead to loss or inactivated enzyme, resulting in accumulation of substrates in lysosomes and increased substrate levels in blood and several organs, that finally cause multiple organ dysfunction, severe pathology and diminished survival. Fabry Disease (FD) is caused by defective alpha-galactosidase A (αGal) that hydrolyses the terminal alpha-galactosyl moiety from glycosphingolipids [56,62]. Enzyme replacement therapies (ERT) have been successfully developed for several LSDs, such as Gaucher (GD), Pompe (PD), and Fabry Disease (FD) [57,58,59]. ERT of FD is presently carried out by intravenous infusion with purified recombinant alpha-galactosidase (α-Gal) in order to increase enzyme levels in lysosomes and reduce substrate accumulation [55,56]. 

Although ERT is effective for a number of LSDs, substantial problems are caused by the formation of antibodies. ERT can trigger the formation of specific antibodies associated with allergic reactions ranging from mild symptoms to anaphylactic shock [54,57]. Moreover, ERT often results in the development of neutralizing IgG antibodies that bind to the infused enzyme and diminish the therapeutic effectiveness [60,61,62]. A substantial part of male FD patients show high IgG antibody titers that cross-react with the recombinant enzyme and neutralize α-Gal activity in up to 95% of patients [54]. During rh-α-Gal A infusion, circulating enzyme–antibody complexes are formed, while IgG-negative patients show a significant reduction in urinary globotriaosylceramide substrates. Neutralizing and cross-reactive antibodies in male FD patients suggested that switching to an alternative enzyme is unfeasible to prevent the immune response. Therefore, alternative treatment approaches by development and application of antibody-specific epitopes capable to block antibodies have been suggested as an alternative for therapeutic intervention [54,62].

**Figure 6:**
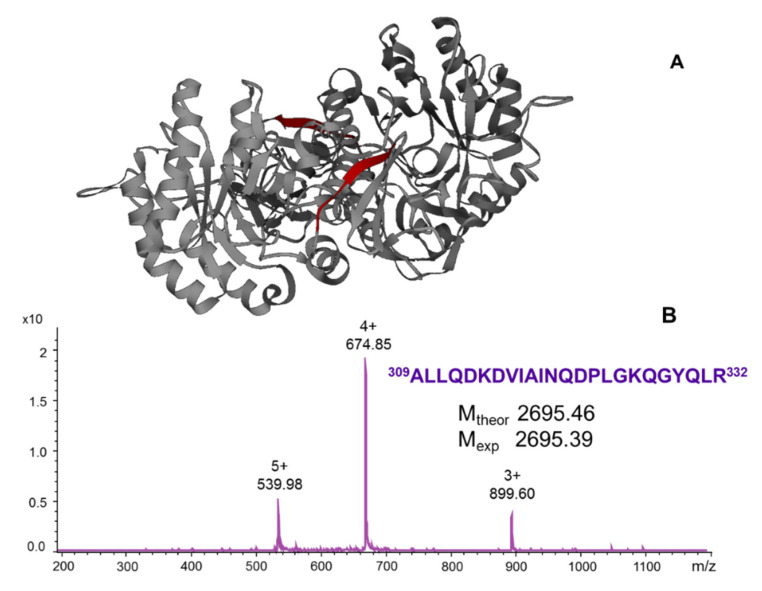
Tertiary structure docking of the αGal (309–322) epitope in the enzyme structure (**A**) and ESI-mass spectrum of the epitope from the affinity elution fraction after α−Gal tryptic digestion (**B**). The *m/z* values of peaks correspond to the charge states of multiply protonated peptide ions [62].

To identify the αGal epitope(s), proteolytic epitope—extraction and - excision—MS were employed using a Sepharose-immobilized anti-α-Gal antibody column and trypsin digestion, and protein G-immobilized serum antibodies. αGal was digested with trypsin and the peptide fragment mixture analyzed as a supernatant and subjected to interaction with the serum antibody. MALDI-MS of the supernatant fractions revealed complete sequence coverage of αGal, except for the short peptide KRK (440–442) consisting of three basic residues. ESI-MS of the elution fraction from the antibody revealed a single epitope peptide, αGal (309–332), with protonated 3+, 4+ and 5+ charged ions (Figure 6). Unequivocal epitope identification was ascertained by MALDI-MS molecular mass and fragment determinations [62,63]. Upon antibody binding; the uncleaved lysine residues K-314 and K-326 suggested their shielding in the antibody interaction. Identical epitope peptides were found in serum antibodies of two FD patients. The epitope peptide (309–332) is illustrated in a tertiary structure docking of the αGal homodimer (Figure 6 and Figure S9), suggesting a defined epitope conformation in the free protein structure; however, structural details upon antibody binding have not yet been determined. Notably, the synthetic peptide epitope αGal (309–332) revealed high affinity, which was comparable to that of the full length enzyme protein. In contrast, partial sequences of the epitope peptide showed substantially lower affinities (Table 2; Figure S9). Although the high affinity found for the αGal (309–332) epitope is only a first observation in the present study, the use of neutralizing epitopes might be a promising future approach for antibody depletion upon ERT.

### 2.6. DNA-Aptamers as Alternative Antibodies: Epitope Determination of an Aptamer-C-Met Protein Complex

The discovery of DNA-aptamers that specifically bind to rat CD4 but not to human CD4 proteins [64,65] initiated high interest in exploring aptamers as new potential diagnostic and therapeutic agents. Aptamers are single stranded DNA or RNA oligonucleotides that exert pronounced selectivity, e.g., as inhibitors for signaling pathways [66,67,68]. In contrast to antibodies, aptamers are chemically synthesized [69,70]; they are obtained by an in vitro selection and optimization procedure termed systematic evolution of ligands by exponential enrichment (SELEX; Figure S10), and exhibit some unique features for the development of bioassays, drug development, and as targeted biomarkers [67,68]. As “chemical antibodies”, aptamers are non-immunogenic and do not interfere with cell viabilities, since they specifically bind and release cells, suggesting high potential for the development of biomarkers. Thus, aptamers reveal a number of potential advantages compared to IgG antibodies, such as fast and easy production, high stabilities and binding strengths [68,70].

The tumor diagnostic protein C-Met is a glycosylated receptor tyrosine kinase of the hepatocyte growth factor (HGF), composed of an alpha- and a beta-chain (Figures S11 and S12). Upon ligand binding and autophosphorylation, C-Met transmits intracellular signals by a multi-substrate docking site; it can be aberrantly activated leading to tumorigenesis and several other diseases and has been recognized as a biomarker in cancer diagnosis [71]. DNA aptamers of C-Met have been considered a promising tool for detection of cancer biomarkers. In a study aimed at the elucidation of inhibiting mechanism(s) for C-Met, DNA aptamers have been explored that bind C-Met with high affinity and specificity (Figure S11) [71]. 

Two aptamers consisting of 60 and 64 bases were synthesized with amino groups (5AmMC12) on the 5′ends [69]. The binding epitopes between C-Met and the aptamers (CLN003 and CLN004) were identified by immobilization of aptamers both on a Sepharose affinity column and a SPR chip, in order to evaluate their binding properties and possible similarities with C-Met antibodies (Figure S11). Affinity determinations of the C-Met- aptamer complexes were performed out by SPR following immobilization of either the aptamers or the protein on a SAM chip, and binding constants were determined with dilution series, respectively. The comparison of the interactions for the two aptamer- C-Met complexes showed two discrete binding steps, respectively, suggesting distinct binding sites [69] (Figure S12). The CLN0003–C-Met interactions were analyzed using two different methods: (i), binding and K_D_ determination using a one to one kinetic model of interaction sites; (ii), affinity analysis and separate determination of the K_D_ of the second binding step. The second binding event was observed after approximately one minute. The corresponding sensorgram showed high affinity with a K_D_ of approximately 54 nM. These results suggested that upon a first interaction step, the aptamer–protein complex undergoes a conformational change that allows a second interaction step to occur [69]. 

**Figure 7:**
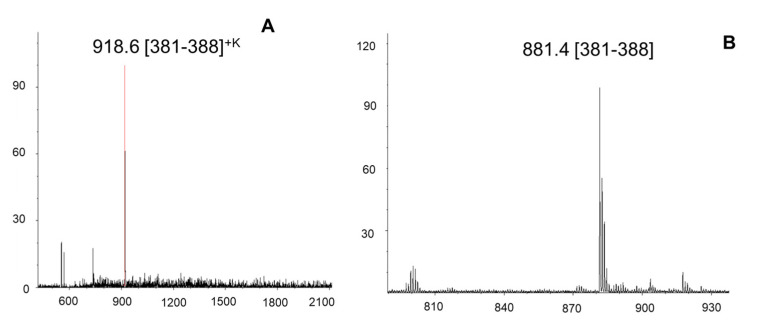
Epitope identification of the complex of the cancer diagnostic protein C-Met with the DNA-aptamer CLN-004 by tryptic epitope extraction from immobilized aptamer on CNBr activated Sepharose affinity column [69]. (**A**); SPR chip-MALDI-MS upon tryptic epitope extraction from immobilized CLN0004 on an SAM-coated SPR chip (**B**), MALDI-MS upon epitope extraction between C-Met and CLN0004 aptamer.

Epitope determinations were performed with the immobilized aptamers using two complementary approaches: (i), Epitope extraction following aptamer immobilization on a micro-affinity column; and (ii), epitope extraction and MALDI-MS following immobilization of aptamers on the SPR chip. Following removal of supernatant peptides by washing with aqueous solvent at binding pH, no background peptide was observed in the last washing fraction. Elution was then performed with slightly acidified solvent containing 0.1% TFA. MALDI-MS of the elution fraction from epitope extraction of C-Met from the CLN0004 aptamer affinity column, and epitope elution from the aptamer-immobilized SPR chip are shown in Figure 7. Both analyses provided a most abundant singly charged epitope peptide, NSSGC(carbamidomethyl)EAR with the sequence (381–388; protonated and Na+ and K+ adduct ions). An additional smaller peptide was found at *m*/*z* 800.4 and identified as RDEYR (389–393). An identical epitope (381–388) was found upon elution from the SPR chip. Thus, a single linear epitope, NSSGCEARRDEYR (C-Met (381–393) was identified for the CLN0004 aptamer. Conversely, the CLN0003–C-Met interaction provided a discontinuous (conformational) epitope comprised of two peptides, C-Met (524–543) and C-Met (557–568) (Figure S13). 

## 3. Conclusions

The combination of biosensor analysis and proteolytic affinity–mass spectrometry described here is expected to play a significant role in the molecular identification and functional characterization of antibody epitopes by mass spectrometry. The major tools, proteolytic excision- and extraction-mass spectrometry (PROTEX-MS), utilize the generation of specific fragments of a protein antigen submitted to an immobilized antibody on the surface of an SPR chip. The identified epitope peptides are characterized by biosensor analysis for affinity quantification, as their key functional properties.

The capability for concomitant affinity quantification and chemical structure identification of epitopes using the PROTEX-MS method meets a considerable need for corresponding analytical tools [6,7,8,27]. No alternative methods have been hitherto reported providing affinity data concomitant with epitope determination. Thus, epitope analysis by HDX-MS does not provide affinity quantification. Likewise, pepscan and related techniques of epitope mapping using synthetic peptides do not provide affinity binding constants, or are feasible to derive affinity data merely in a semi-quantitative fashion.

The efficiency of the PROTEX-MS combination technology has been established in a wide range of biochemical and biomedical application areas, and in first clinical applications for epitope identification of antibody-protein complexes (e.g., [7,62]). Successful applications have been shown both for monoclonal antibody epitopes, and immunodominant epitopes of polyclonal antibodies. Moreover, several studies have shown the advantageous use of protein G immobilization by binding to F_c_ antibody structures, as illustrated in clinical studies on therapeutic antibodies, as well as pathophysiological antibodies formed upon protein therapy [62]. Thus, the epitope peptide for an antibody of α-Galactosidase A used for enzyme replacement therapy of Fabry Disease showed high affinity (K_D_ < 50 nM), comparable to that of an antibody to the therapeutic enzyme [62]. The identification of high affinity epitopes of neutralizing antibodies in ERT of lysosomal diseases is opening a new concept for therapeutic intervention using synthetic peptides for molecular apheresis [57,58]. Although therapeutic proteins, such as recombinant enzymes in ERT, are often highly effective, the formation of antibodies that trigger adverse immunological reactions is currently an unsolved problem, in causing antibody formation against the infused enzyme. Since the extent of antibody formation and severity of pathophysiological effects is often especially high in patients with complete lack of expression of the enzyme, the application of epitope peptides with neutralizing effect to antibodies may be a promising clinical alternative [62]. 

In summary, the combination of bioaffinity and mass spectrometry technologies bears high potential for the development of “hybrid” tools of affinity and chemical structure determination of biomolecular interactions. Although biosensor technologies alone have principal limitations for providing chemical structure information of protein–ligand interactions, mass spectrometry technologies using selective shielding and protection by proteolytic excision and extraction constitute a broad molecular platform for the analysis of biomolecular interactions. The PROTEX-MS technology has been shown to be well applicable to different types of antibodies (e.g., single chain antibodies); it is feasible for combination with different biosensor techniques, and can be combined with other techniques, such as selective chemical modification [72,73,74,75,76,77,78]. Moreover, the recent discovery that DNA aptamers exhibit protein epitopes of comparable specificity and affinity may develop as an attractive alternative to protein antibodies [68,69,70]. Aptamers as non-protein materials could be valuable in applications of proteolytic affinity-mass spectrometry; hence, their evaluation as biomolecular recognition epitopes to proteins may be of high interest in future studies. In summary, the integration of biosensor and mass spectrometry is opening a new hybrid technology with the potential to provide substantial progress for the discovery and validation of diagnostic biomarkers, drug development, and therapeutic lead structures. 

## 4. Methods and Experimental Techniques

### 4.1. Materials and Proteins

Myoglobin, native heme protein and apoprotein from equine heart (purity ≥90% by SDS-PAGE) and human MG, essentially salt-free, lyophilized powder were obtained from Sigma-Aldrich, Hamburg, Germany. Stock solutions of MG of 1 mg/mL were prepared in PBS at a 1:40 ratio of enzyme to protein. Interleukin-8 (IL8) and a murine monoclonal antibody (IgG1) from a hybridoma cell line were obtained from Sigma-Aldrich. Human recombinant TNF-α (soluble fraction (77–233)) was obtained from Abcam, Wiesbaden, Germany. Recombinant lysosomal enzyme samples and antibodies were obtained from Sigma-Aldrich and Sanofi-Genzyme, Frankfurt, Germany. Recombinant Human C-Met protein/Fc Chimera was obtained from Sino Biological (Wayne, PA, USA). The protein comprised the extracellular domain of human C-Met (Met-1 to Thr-932) fused to a C-terminal poly-His-tagged Fc domain of human IgG1.

DNA aptamers (CLN0003 and CLN0004) were prepared by IDT Integrated Technologies (Coralville, IA, USA) (Figure S11).

### 4.2. Proteolytic Digestion 

Protein reduction and alkylation prior to proteolytic digestion was performed at standard conditions as previously reported [31,40,49,60]. A solution of 20 µg protein in 40 mM NaH_2_PO_4_ containing 35 mM NaCl (pH 7.4) was diluted with 40 µL 100 mM ammonium hydrogen carbonate, and mixed with a 10 mM solution of dithiotreitol (DTT; 3 µL in MilliQ-water). After incubation for 15 min at 95 °C and cooling to 20 °C, 6 µL of 100 mM iodoacetamide (IAA) in water was added and incubated for 60 min in the dark. Subsequently, a 3 µL aliquot of DTT solution (100 mM) was added and the reaction mixture incubated for further 30 min in the dark. 

Standard conditions for digestion at atmospheric pressure were employed with a 1 µL aliquot of a solution (0.5 mg/mL) of trypsin (Promega, Mannheim, Germany; sequencing grade) added to an aliquot of 20 µg protein(s), and the reaction mixture incubated for 18 h at 37 °C. High pressure digestion was performed with a Barocycler 2320GTX instrument (Pressure Biosciences Inc., Boston, MA, USA). Proteins in the high pressure cell were set to undergo pressure cycles of 50 s at 20 kpsi, and subsequent 10 s pressure release to atmospheric pressure. Typically, 90–120 cycles were executed at 37 °C for complete digestion. Prolonged times and increased pressure were applied for proteins showing slow unfolding (such as myoglobins), for which digestion times of 120–400 cycles were used. Epitope determinations using proteolytic extraction with tryptic digestion mixtures were performed both following high pressure and atmospheric pressure digestion. For C-Met protein, proteolytic digestion was performed both at atmospheric pressure conditions and at high pressure. Aliquots of 10 µg C-Met in 15 µL ammonium hydrogen carbonate (100 mM, pH 7.6) were subjected to reduction and alkylation prior to proteolytic digestion, as previously described [60].

### 4.3. Immobilization Procedures of Antibodies and Aptamers

Immobilization of antibodies and aptamers on CNBr-activated 4B Sepharose columns was performed according to a similar procedure for all affinity pairs studied. Matrix preparation was performed by incubation in 0.1 M HCl for 15 min, followed by washing with coupling buffer (0.2 M sodium hydrogen carbonate + 0.5 M NaCl, pH 8). Samples (30–100 µg of antibody) were incubated by shaking for 2–3 h. Antibody columns were washed alternatively with washing buffer (0.2 M sodium acetate + 0.5 M NaCl, pH 4) and blocking buffer (1 M Ethanolamine + 0.5 M NaCl, pH 8). Column preparation was completed by incubation with blocking buffer for 2–18 h.

Aptamer immobilization was performed in the same manner as for direct antibody immobilization. The aptamer sequences carried a 5’-amino C6 linker that allows the identical immobilization procedure as for antibodies. Samples of 75 µg of aptamers were immobilized, and affinity columns blocked with 0.2 M Ethanolamine + 0.5 M NaCl, pH 8.3, followed by triplicate washing with washing buffer (0.2 M sodium acetate + 0.5 M NaCl, pH4) and were resuspended in ammonium hydrogen carbonate buffer, pH 7.5. Affinity columns were stored at 4 °C until use.

For SPR chip immobilization, chip surfaces were washed three times with alternating water and 70% aqueous ethanol and then immersed for 15 s in Piranha solution (H_2_SO_4_: H_2_O_2_, 2:1). This step was repeated three times, and the cleaned chip incubated overnight at room temperature with shaking in a solution of 16-mercaptohexadodecanoic acid in chloroform and then dried under N_2_. SPR chip immobilization was performed with an Ametek-Reichert 2Ch7500 SPR instrument (Ametek Inc., Buffalo, NY, USA). Preparation of activated SAM chips was performed with a mixture of 200 mM N-(3-dimethylaminopropyl)-N-ethylcarbodiimide (EDC) and 50 mM N-hydroxysuccinimide (NHS), followed by injection of 10 to 50 µg antibody or aptamer in 250 µL 10 mM sodium acetate, pH 5.5. Remaining free NHS reagent was blocked with 1 M ethanolamine. 

Two strategies were used to immobilize antibodies on the chip surface: (i), direct, random immobilization by surface activation with a mixture of EDC (40 mg/mL) and NHS (10 mg/mL). After immobilization of 40 µg antibody, the chip is blocked with 1 M ethanolamine (pH 8.5) in water at 25 µL/min for 10 min; (ii), in a second approach, oriented antibody immobilization is performed by surface activation with EDC (40 mg/mL) and NHS (10 mg/mL) at a flow rate of 25 µL/min for 8 min, followed by addition of 50 µg protein G (0.2 µg/µL in 10 mM sodium acetate buffer, pH 5.2) immobilized at 25 µL/min for 10 min, and additional capping with 1 M ethanolamine (pH 8.5) for 10 min. Subsequently, 1–5 µg antibody (0.004–0.02 µg/µL in PBS, pH 7.4) is incubated with protein-G at 10 µL/min for 25 min. Using the same flow rate, 30 mM dimethylpimelidate (DMP) in 200 mM sodium borate (pH 9) is flushed over the antibody–protein G complex, and capping performed with 1 M ethanolamine (pH 8.5).

A POROS G sensor cartridge containing covalently immobilized protein G was also used. In this case, antibodies from patient sera were captured on the column and crosslinked as described above. Prior to epitope extraction, the column was equilibrated with buffer (10 mM NaHPO_4_ and 150 mM NaCl, pH 7.5) for 10 min. Patients sera upon ERT were stored refrigerated until use, and centrifuged at 4000× *g* prior to application. For affinity capture of IgG antibodies, 2.5 mL of serum was injected on the cartridge at a flow rate of 0.5 mL/min. The flow through was monitored at 280 nm with a UV/VIS detector and collected. In order to block remaining protein G, 500 µL of diluted human IgG fraction was injected on the column.

### 4.4. SPR Biosensor Techniques

SPR Analyses were performed with an Ametek-Reichert 2Ch7500 SPR instrument (Ametek-Reichert, Buffalo, NY, USA). Standard SPR gold chips (9 × 9 × 0.3 mm) (Sofchip Inc., Sebring, FL, USA), were coated with self-assembled monolayer (SAM) of 16-mercaptohexadecanoic acid. The SPR cell consisting of two channels (one of which containing the immobilized sample) provides the convenient determination of SPR sensorgrams, e.g., using the TraceDrawer 1.7.1 software from Ridgeview Instruments (Vange, Sweden). 

### 4.5. Epitope Determination by Proteolytic Extraction MS

Epitope determination from affinity columns was performed by proteolytic epitope extraction MS. Following the mass spectrometric characterization of proteolytic digestion mixtures, peptides are incubated with the antibody-bound or aptamer-bound affinity column for 2–3 h. Supernatant unbound peptides are removed with washing buffer and subsequently analyzed by MALDI-MS and compared to the initial digestion mixtures; washing is typically continued until the mass spectra show no residual background signal (usually 20 column volumes). Following mass spectrometric background analysis, the affinity columns are subjected to slight acidification (0.01% TFA) and epitope(s) peptide(s) eluted (typically 2 column elution volumes in 0.1% TFA). After re-equilibration with washing buffer, the column is stored at 4 °C. Collected fractions are centrifuged in vacuo (Eppendorf Concentrator) until the volume is reduced to 10–30 µL. MALDI-MS Determination of elution fractions was performed using DHB as a matrix. 

### 4.6. Epitope Analyses from SPR-Chips

Direct proteolytic epitope extraction on the SPR chip was performed with running buffer set to 50 mM ammonium bicarbonate buffer (typically at a flow rate of 5 µL/min), and a 20 µg aliquot of the tryptic digestion sample injected over the immobilized antibody/aptamer. Subsequently the chip is flushed with buffer (60–120 min) and fractions collected over a period of 10–20 min. Elution is performed by injection of 0.1% TFA in water, and each fraction concentrated to 10–30 µL prior to MALDI-MS.

### 4.7. Mass Spectrometry

MALDI-TOF-MS was performed with a Bruker Autoflex-III Smartbeam (Bruker Daltonics, Bremen, Germany). For protein analyses a 50 mg/mL SDHB matrix solution (Super DHB; Bruker Daltonics, Bremen, Germany) in 50:50 [*v*/*v*] acetonitrile: 0.1% TFA in MilliQ-H_2_O was used. Epitope peptide fractions and synthetic peptides were analyzed with a 20 mg/mL 2,5-DHB matrix solution in 50:50 acetonitrile: 0.1% TFA matrix. Typically, 0.5 µL of sample is spotted on the stainless steel MALDI sample target and mixed on the target with 0.5 µL of matrix. 

### 4.8. Synthesis and Affinity Characterization of Epitope Peptides

Peptide syntheses (Fmoc-SPPS) were carried out with an automated Applied Biosystems peptide synthesizer ABI-433. Epitope peptides were synthesized on preloaded PS-PHB resins with the corresponding amino acids at the C-terminus. Standard syntheses of 0.1 mmol for each peptide is performed using Fmoc protected amino acids. Side chain protection and removal of peptides from the resin are carried out by incubating the resin for 1 h in 5 mL of 94% TFA (with 3% TIS and 3% water). The crude peptides precipitated into 20 mL diethyl ether are centrifuged and the diethyl ether decanted. Purification of peptides was performed by HPLC using a RP-C18 column (250 × 4.6 mm), and characterization for molecular homogeneity is carried out by ESI-MS and/or MALDI-MS.

The 23aa αGal epitope peptide (309–332) and the IL8 epitopes (12–20 and 55–60) were synthesized as described above [27,40], while αGal partial epitope peptides ((309–314), (315–326), (327–332)) were synthesized on a semiautomatic peptide synthesizer (ResPepSL; Intavis Bioanalytical Instruments, Cologne, Germany).

## Figures and Tables

**Table 1:** Biomolecular binding affinities of horse heart MG determined by SPR.

Method	Random K_D_ (µM) (a)	Protein G K_D_ (µM) (b)
Kinetics evaluation	0.028	0.121
Affinity plot	0.280	0.145
Mean Value with error	0.154 ± 0.126	0.133 ± 0.12

^a^ K_D_ determination with undetermined orientation of immobilized Antibody. ^b^ K_D_ determination with protein G immobilization strategy.

**Table 2:** Binding affinities of full length α-Gal protein and α-Gal epitope peptides determined by SPR.

	Protein/Peptide	K_D_ (µM)
Intact protein	α-Gal (1–429)	0.015
Epitope	α-Gal (309–332)	0.038
Peptide I	α-Gal (309–320)	151
Peptide II	α-Gal (315–326)	43.4
Peptide III	α-Gal (321–332)	7.05
Peptide IV	α-Gal (309–316)	816
Peptide V	α-Gal (317–324)	1600
Peptide VI	α-Gal (325–332)	493

## Data Availability

Published data in this article are available in public repositories, in Wiegand P., Lupu L., Hüttmann N., Wack J., Rawer S., Przybylski M., Schmitz K., Epitope Identification and Affinity Determination of an Inhibiting Human Antibody to Interleukin IL8 (CXCL8) by SPR-Biosensor–Mass Spectrometry Combination, J. Am. Soc. Mass Spectrom., 2020, 31, 1, 109–116, doi:10.1021/jasms.9b00050; Lupu L., Wiegand P., et al. ChemMedChem, 2020, 15, 4, 363–369, doi:10.1002/cmdc.201900489; Mihoc D., Lupu L.M., et al., J. Am. Soc. Mass Spectrom., 2021, 32, 1, 106–113, doi:10.1021/jasms.0c00234. Other data due to restrictions by privacy are available on request from the corresponding author.

## Supplementary Materials

The following Supplementary Materials are available online at https://www.mdpi.com/article/10.3390/ijms222312832/s1.

## References

[1] Ecker D.M., Jones S.D., Levine H.L. (2015). The therapeutic monoclonal antibody market. mAbs.

[2] Aggarval S.R. (2014). A survey of breakthrough therapy designations. Nat. Biotechnol..

[3] Walsh G. (2014). Biopharmaceutical benchmarks. Nat. Biotechnol..

[4] Van Regenmortel M.H.V. (2014). Specificity, polyspecificity, and heterospecificity of antibody-antigen recognition. J. Mol. Recognit..

[5] Barlow D.J., Edwards M.S., Thornton J.M. (1986). Continuous and discontinuous protein antigenic determinants. Nature.

[6] Przybylski M., Gauglitz G., Moore D.S. (2014). Mass spectrometry. Hand-Book of Spectroscopy.

[7] Opuni K.F.M., Al-Majdoub M., Yefremova Y., El-Kased R.F., Koy C., Glocker M.O. (2018). Mass Spectrometric Epitope Mapping. Mass Spectrom. Rev..

[8] Petre B.A., Ulrich M., Stumbaum M., Bernevic B., Moise A., Döring G., Przybylski M. (2012). When is mass spectrometry combined with affinity approaches essential? A case study of tyrosine nitration in proteins. J. Am. Soc. Mass Spectrom..

[9] Dragusanu M., Petrel B.A., Slamnoiu S., Vlad C., Tu T., Przybylski M. (2010). Online bioaffinity-electrospray mass spectrometry for simultaneous detection, identification, and quantification of protein-ligand interactions. J. Am. Soc. Mass Spectrom..

[10] Hager-Braun C., Tomer K.B. (2005). Determination of protein-derived epitopes by mass spectrometry. Expert Rev. Proteom..

[11] Dhungana S., Williams J.G., Fessler M.B., Tomer K.B., Reineke U., Schutkowskim M. (2009). Epitope mapping by proteolysis of antigen-antibody complexes. Epitope Mapping Protocols.

[12] Defaus S., Avilés M., Andreu D., Gutiérrez-Gallego R. (2018). Lectin-Binding Specificity of the Fertilization-Relevant Protein PDC-109 by Means of Surface Plasmon Resonance and Carbohydrate Recognition Domain Excision-Mass Spectrometry. Int. J. Mol. Sci..

[13] Paterson Y., Englander S.W., Roder H. (1990). An antibody binding site on cytochrome c defined by hydrogen exchange and two-dimensional NMR. Science.

[14] Malito E., Carfi A., Bottomley M.J. (2015). Protein Crystallography in Vaccine Research and Development. Int. J. Mol. Sci..

[15] Carter J.M. (2016). Epitope mapping of a protein using the Geysen (PEPSCAN) procedure. Methods Mol. Biol..

[16] Kristensen C., Kjeldsen T., Wiberg F.C., Schäffer L., Hach M., Havelund S., Bass L., Steiner D.F., Andersen A.S. (1997). Alanine Scanning Mutagenesis of Insulin. J. Biol. Chem..

[17] Zhu C., Liu X., Feng J., Zhang W., Shen B., Ou’yang W., Cao Y., Jin B. (2006). Characterization of the neutralizing activity of three anti-human TNF monoclonal antibodies and prediction of their TNF epitopes by molecular modeling and mutant protein approach. Immunol. Lett..

[18] Chen Y., Wiesmann C., Fuh G., Li B., Christinger H.W., McKay P., deVos A.M., Lowman H.B. (1999). Selection and analysis of an optimized anti-VEGF antibody: Crystal structure of an affinity-matured Fab in complex with antigen. J. Mol. Biol..

[19] Engen J.R., Wales T.E. (2015). Analytical Aspects of Hydrogen Deuterium Mass Spectrometry. Ann. Rev. Anal. Chem..

[20] Pirrhone G.F., Vernon B.C., Kent M.S., Engen J.R. (2015). Hydrogen Deuterium Exchange Mass Spectrometry of Proteins at Langmuir Monolayers. Anal. Chem..

[21] Chen J., Rempel D.L., Gau B.C., Gross M. (2012). Fast Photochemical Oxidation of Proteins and Mass Spectromety follow submillisecond Protein Folding at the Amino Acid Level. J. Am. Chem. Soc..

[22] Gau B., Chen J., Gross M.L. (2013). Fast Photochemical Oxidation of Proteins for Comparing Solvent-accessible Changes Accompanying Protein Folding. Biochim. Biophys. Acta.

[23] Narang D., Lento C., Wilson J.D. (2020). HDX-MS: An Analytical Tool to Capture Protein Motion in Action. Biomedicines.

[24] Jones M.L., Legge F.S., Lebani K., Mahler S.M., Young P.R., Watterson D., Treutlein H.R., Zeng J. (2017). Computational Identification of Antibody Epitopes on the Dengue Virus NS1 Protein. Molecules.

[25] Demolombe V., de Brevern A.G., Molina F., Lavigne G., Granier C., Moreau V. (2019). Benchmarking the PEPOP methods for mimicking discontinuous epitopes. BMC Bioinform..

[26] Suckau D., Kohl J., Karwath G., Schneider K., Casaretto M., Bitter-Suermann D., Przybylski M. (1990). Molecular epitope identification by limited proteolysis of an antigen-antibody complex and mass spectrometric peptide mapping. Proc. Natl. Acad. Sci. USA.

[27] Stefanescu R., Iacob R.E., Damoc E.N., Marquardt A., Amstalden E., Manea M., Perdivara I., Maftei M., Paraschiv G., Przybylski M. (2007). Mass spectrometric approaches for elucidation of antigen-antibody recognition structures in molecular immunology. Eur. J. Mass Spectrom..

[28] Legros V., Jolivet-Reynaud C., Battail-Poirot N., Saint-Pierre C., Forest E. (2000). Characterization of an anti-Borrelia burgdorferi OspA conformational epitope by limited proteolysis of monoclonal antibody-bound antigen and mass spectrometric peptide mapping. Protein Sci..

[29] McLaurin J., Cecal R., Kierstead M.E., Tian X., Phinney A.L., Manea M., French J.E., Lambermon M.H., Darabie A.A., Brown M.E. (2002). Therapeutically effective antibodies against amyloid-beta peptide target amyloid-beta residues 4-10 and inhibit cytotoxicity and fibrillogenesis. Nat. Med..

[30] Juszczyk P., Paraschiv G., Szymanska A., Kolodziejczyk A.S., Rodziewicz-Motowidlo S., Grzonka Z., Przybylski M. (2009). Binding epitopes and interaction structure of the neuroprotective protease inhibitor cystatin C with beta-amyloid revealed by proteolytic excision mass spectrometry and molecular docking simulation. J. Med. Chem..

[31] Iurascu M.I., Marroquin Belaunzanar O., Cozma C., Petrausch U., Renner C., Przybylski M. (2016). An HLA-B27 Homodimer Specific Antibody Recognizes a Discontinuous Mixed- Disulfide Epitope as Identified by Affinity-Mass Spectrometry. J. Am. Soc. Mass Spectrom..

[32] Paraschiv G., Vincke C., Czaplewska P., Manea M., Muyldermans S., Przybylski M. (2013). Epitope structure and binding affinity of single chain llama anti-β-amyloid antibodies revealed by proteolytic excision affinity-mass spectrometry. J. Mol. Recognit..

[33] Moise A., André S., Eggers F., Krzeminski M., Przybylski M., Gabius H.J. (2011). Toward bioinspired galectin mimetics: Identification of ligand-contacting peptides by proteolytic-excision mass spectrometry. J. Am. Chem. Soc..

[34] Stefanescu R., Born R., Moise A., Ernst B., Przybylski M. (2011). Epitope structure of the carbohydrate recognition domain of asialoglycoprotein receptor to a monoclonal antibody revealed by high-resolution proteolytic excision mass spectrometry. J. Am. Soc. Mass Spectrom..

[35] Lakayan D., Haselberg R., Gahoual R., Somsen G.W., Kool J. (2018). Affinity profiling of monoclonal antibody and antibody-drug-conjugate preparations by coupled liquid chromatography-surface plasmon resonance biosensing. Anal. Bioanal. Chem..

[36] Florinskaya A., Ershov P., Mezentsev Y. (2018). SPR biosensors in direct molecular Fishing: Implications for Protein Interactomics. Sensors.

[37] Bouffartigues E., Leh H., Anger-Leroy M., Rimsky S., Buckle M. (2007). Rapid coupling of Surface Plasmon Resonance (SPR and SPRi) and ProteinChip based mass spectrometry for the identification of proteins in nucleoprotein interactions. Nucleic Acids Res..

[38] Mihoc D., Lupu L.M., Wiegand P., Kleinekofort W., Müller O., Völklein F., Glocker M.O., Barka F., Barka G., Przybylski M. (2021). Antibody Epitope and Affinity Determination of the Myocardial Infarction Marker Myoglobin by SPR-Biosensor Mass Spectrometry. J. Am. Soc. Mass Spectrom..

[39] Atassi M.Z., Tarlowski D.P., Paull J.H. (1970). Immunochemistry of sperm whale myoglobin, vll. Correlation of immunochemical cross-reaction of eight myoglobins with structural similarity and its dependence on conformation. Biochim. Biophys. Acta Protein Struct..

[40] Berkower I., Buckenmeyer G.K., Gurd F.R., Berzofsky J.A. (1982). A possible immunodominant epitope recognized by murine T lymphocytes immune to different myoglobins. Proc. Natl. Acad. Sci. USA.

[41] Macht M., Fiedler W., Kuerzinger K., Przybylski M. (1996). Mass Spectrometric Mapping of Protein Epitope Structures of Myocardial Infarct Markers Myoglobin and Troponin, T. Biochemistry.

[42] Bjorck L., Kronvall G. (1984). Purification and some properties of streptococcal protein G, a novel IgG-binding reagent. J. Immunol..

[43] Chen P.K., Lan J.L., Chen Y.M., Chen H.H., Chang S.H., Chung C.M., Rutt N.H., Tan T.M., Mamat R.N.R., Anuar N.D. (2021). Anti-TROVE2 Antibody Determined by Immune-Related Array May Serve as a Predictive Marker for Adalimumab Immunogenicity and Effectiveness in RA. J. Immunol. Res..

[44] Homann A., Röckendorf N., Kromminga A., Frey A., Platts-Mills T.A., Jappe U. (2017). Glycan and Peptide IgE Epitopes of the TNF-alpha Blockers Infliximab and Adalimumab—Precision Diagnostics by Cross-Reactivity Immune Profiling of Patient Sera. Theranostics.

[45] Van Schouwenburg P.A., Kruithof S., Votsmeier C., van Schie K., Hart M.H., de Jong R.N., van Buren E.E., van Ham M., Aarden L., Wolbink G. (2014). Functional analysis of the anti-adalimumab response using patient-derived monoclonal antibodies. J. Biol. Chem..

[46] Krayukhina E., Noda M., Ishii K., Maruno T., Wakabayashi H., Tada M., Suzuki T., Ishii-Watabe A., Kato M., Uchiyama S. (2017). Analytical ultracentrifugation with fluorescence detection system reveals differences in complex formation between recombinant human TNF and different biological TNF antagonists in various environments. mAbs.

[47] Suzuki M., Ozawa F., Sugimoto W., Aso S. (2002). Miniature surface-plasmon resonance immunosensors-rapid and repetitive procedure. Anal. Bioanal. Chem..

[48] Bergström G., Mandenius C.F. (2011). Orientation and capturing of antibody affinity ligands: Applications to surface plasmon resonance biochips. Sens. Actuators B.

[49] Rusche H. (2017). Identification of Adalimumab Epitopes Recognized by Anti-drug Antibodies. Master’s Thesis.

[50] Lowman H.B., Fairbrother W.J., Slagle P.H., Kabakoff R., Liu J., Shire S., Hebert C.A. (1997). Monomeric variants of IL-8: Effects of side chain substitutions and solution conditions upon dimer formation. Protein Sci..

[51] Helmer D., Rink I., Dalton J.A.R., Brahm K., Jöst M., Nargang T.M., Blum W., Wadhwani P., Brenner-Weiss G., Rapp B.E. (2015). Rational design of a peptide capture agent for CXCL8 based on a model of the CXCL8:CXCR1 complex. RSC Adv..

[52] Clubb R.T., Omichinski J.G., Clore G.M., Gronenborn A.M. (1994). Mapping the binding surface of interleukin-8 complexed with an N-terminal fragment of the Type 1 human interleukin-8 receptor. FEBS Lett..

[53] Wiegand P., Lupu L., Huettmann N., Wack J., Rawer S., Przybylski M., Schmitz K. (2020). Epitope Identification and Affinity Determination of an Inhibiting Human Antibody to Interleukin IL8 (CXCL8) by SPR- Biosensor—Mass Spectrometry Combination. J. Am. Soc. Mass Spectrom..

[54] Bigger B.W., Saif M., Linthorst G.E. (2015). The role of antibodies in enzyme treatments and therapeutic strategies. Best Pract. Res. Clin. Endocrinol. Metab..

[55] Ortolano S., Vieitez I., Navarro C., Spuch C. (2014). Treatment of Lysosomal Storage Diseases: Recent Patents and Strategies. Rec. Pat. Endocr. Metab. Immune Drug Discov..

[56] Desnik R.J. (2004). Enzyme replacement therapy for Fabry disease: Lessons from two galactosidase A orphan products and one FDA approval. Expert Opin. Biol. Ther..

[57] Banugaria S.N., Prater S.N., Ng Y.K., Kobori J.A., Finkel R.S., Ladda R.L., Chen Y.T., Rosenberg A.S., Kishani P.S. (2011). The impact of antibodies on clinical outcomes in diseases treated with therapeutic protein: Lessons learned from infantile Pompe disease. Genet. Med..

[58] Banugaria S.G., Patel T.T., Mackey J., Das S., Amalfitano A., Rosenberg A.S., Charrow J., Chen Y.T., Kishani P.S. (2012). Persistence of high sustained antibodies to enzyme replacement therapy despite extensive immunomodulatory therapy in an infant with Pompe disease: Need for agents to target antibody secreting plasma cells. Mol. Genet. Metab..

[59] Valaianopoulos V., Wijburg F.A. (2011). Therapy for the Mucopolysaccharidoses. Rheumatology.

[60] Hennermann J.B., Gökce S., Solyom A., Mengel E., Schuchman E.H., Simonaro C.M. (2016). Treatment with pentosan polysulphate in patients with MPS I: Results from an open label, randomized, monocentric phase II study. J. Inherit. Metab. Dis..

[61] Langereis E.J., van Vlies N., Church H.J., Geskus R.B., Hollak C.E., Jones S.A., Kulik W., van Lenthe H., Mercer J., Schreider L. (2015). Biomarker responses correlate with antibody status in mucopolysaccharidosis type I patients on long-term enzyme replacement therapy. Mol. Genet. Metab..

[62] Kukacka Z., Iurascu M., Lupu L., Rusche H., Murphy M., Altamore L., Borri F., Maeser S., Papini A.M., Hennermann J. (2018). Antibody Epitope of Human alpha-Galactosidase A Revealed by Affinity Mass Spectrometry: A Basis for Reversing Immunoreactivity in Enzyme Replacement Therapy of Fabry Disease. ChemMedChem.

[63] Moise A., Maeser S., Rawer S., Eggers F., Murphy M., Bornheim J., Przybylski M. (2016). Substrate and Substrate-Mimetic Chaperone Binding Sites in Human alpha-Galactosidase A Revealed by Affinity-Mass Spectrometry. J. Am. Soc. Mass. Spectrom..

[64] Zamay T.N., Zamay G.S., Kolovskaya O.S., Zukov R.A., Petrova M.M., Gargaun A., Berezovski M.V., Kichkailo A.S. (2017). Current and Prospective Protein Biomarkers of Lung Cancer. Cancers.

[65] Zamay G.S., Ivanchenko T.I., Zamay T.N., Grigorieva V.L., Glazyrin Y.E., Kolovskaya O.S., Garanzha I.V., Barinov A.A., Krat A.V., Mironov G.G. (2017). DNA Aptamers for the Characterization of Histological Structure of Lung Adenocarcinoma. Mol. Ther. Nucleic Acids.

[66] Xie S., Walton S.P. (2010). Development of a dual-aptamer-based multiplex protein biosensor. Biosens Bioelectron..

[67] Sabri M.Z., Abdul Hamid A.A., Sayed Hitam S.M., Abdul Rahim M.Z. (2019). In Silico Screening of Aptamers Configuration against Hepatitis B Surface Antigen. Adv. Bioinform..

[68] Labib M., Zamay A.S., Muharemagic D., Chechik A.V., Bell J.C., Berezovski M.V. (2012). Electrochemical differentiation of epitope-specific aptamers. Anal. Chem..

[69] Lupu L., Wiegand P., Hüttmann N., Rawer S., Kleinekofort W., Shugureva I., Kichkailo A.S., Tomilin F.N., Lazarev A., Berezovski M.V. (2020). Molecular Epitope Determination of Aptamer Complexes of the Multidomain Protein C-Met by Proteolytic Affinity-Mass Spectrometry. ChemMedChem.

[70] Famulok M., Mayer G. (2014). Aptamers and SELEX in Chemistry & Biology. Chem. Biol..

[71] Bouattour M., Raymond E., Qin S., Cheng A.L., Stammberger U., Locatelli G., Faivre S. (2018). Recent developments of C-Met as a therapeutic target in hepatocellular carcinoma. Hepatology.

[72] Al-Majdoub M., Koy C., Lorenz P., Thiesen H.J., Glocker M.O. (2013). Mass spectrometric and peptide chip characterization of an assembled epitope: Analysis of a polyclonal antibody model serum directed against the Sjogren/systemic lupus erythematosus autoantigen TRIM21. J. Mass Spectrom..

[73] Ansong C., Miles S.M., Fay P.J. (2006). Epitope mapping factor VIII A2 domain by affinity-directed mass spectrometry: Residues 497-510 and 584-593 comprise a discontinuous epitope for the monoclonal antibody R8B12. J. Thromb Haemost..

[74] El-Kased R.F., Koy C., Deierling T., Lorenz P., Qian Z., Li Y., Thiesen H.J., Glocker M.O. (2009). Mass spectrometric and peptide chip epitope mapping of rheumatoid arthritis autoantigen RA33. Eur. J. Mass Spectrom..

[75] Glocker M.O., Nock S., Sprinzl M., Przybylski M. (1998). Characterization of surface topology and binding area in complexes of the elongation factor proteins EF-Ts and EF-Tu.GDP from thermus thermophilus: A study by protein chemical modification and mass spectrometry. Chem. Eur. J..

[76] Macht M., Marquardt A., Deininger S.O., Damoc E., Kohlmann M., Przybylski M. (2004). Affinity-proteomics: Direct protein identification from biological material using mass spectrometric epitope mapping. Anal. Bioanal. Chem..

[77] Obungu V.H., Gelfanova V., Huang L. (2013). Epitope mapping of antibodies by mass spectroscopy: A case study. Methods Mol. Biol..

[78] Sinz A. (2006). Chemical cross-linking and mass spectrometry to map threedimensional protein structures and protein-protein interactions. Mass Spectrom. Rev..

