# Supplementary material for: Identification and Affinity Determination of Protein-Antibody and Protein-Aptamer Epitopes by Biosensor-Mass Spectrometry Combination
Source: Int J Mol Sci. 2021 Nov 27;22(23):12832. doi: 10.3390/ijms222312832 (PMC8657952; doi:10.3390/ijms222312832)

International Journal of *Molecular Sciences*

MDPI

Supplementary Figures

Figure S1:
Schematic representation of epitope extraction via SPR chip in combination with MALDI MS. Sample injection over the SPR gold chip with the antibody immobilized on one channel. Peptides are injected over the antibody channel allowing the SPR sensorgram recording of the binding peptides and the MALDI-MS of the eluted epitope peptides. The K_D of the intact protein is measured by injecting a dilution series of the intact protein only over the SPR chip.
Figure S2:
Schematic representation of epitope identification by epitope excision–mass spectrometry. After the protein–antibody complex is formed, a protease is added to the complex. The epitope peptide is shielded by the antibody from proteolytic digestion, and can be eluted after washing of non-binding peptides.
Figure S3:
Affinity captured antibody for column preparation with Sepharose immobilized protein G crosslinked to the Fc antibody region.
Figure S4:
Mass spectrometric determination of intact apomyoglobin, average mass 16952.49 Da. A. MALDI-MS of singly, doubly and triply charged protein. B. ESI-MS of multiply charged intact protein.
Figure S5:
MALDI-MS measurements of digested myoglobin. A. MALDI-MS spectra of myoglobin tryptic peptides digested at atmospheric pressure for 18 hours. B. MALDI-MS spectra of tryptic peptides digested with cyclic high pressure technology for 2 hours.
Figure S6:
Crystal structure of Adalimumab Fab (blue and green) in complex with TNF-α(orange). The identified epitope (Adalimumab heavy chain 12-29) is marked in red.
Figure S7:
IL8 3D structure depicting epitope identification. (A) Epitope peptides [12–20] and [55–60] highlighted (red) in the 3D-structure of IL8. (B) Binding sites of IL8 (blue) to CXCR1. (C) Overlapping region of the antibody epitope and the receptor binding site on IL8.
Figure S8:
Epitope extraction applied to IL8 and anti-IL8 antibody. (A) Experimental procedure of the SPR based epitope extraction. (B) Experimental procedure of the micro affinity column based epitope extraction.
Figure S9:
SPR sensorgram of the interaction between intact α-Galactosidase (A)/epitope peptide [309–332] (B) and anti-α-Galactosidase antibody. K_D calculation was performed from three concentrations of intact α-Galactosidase; B, K_D calculation from four concentrations of epitope peptide [309-332] .
Figure S10:
Schematic representation of the SELEX procedure for preparation of affinity-optimized
DNA-aptamers.
Figure S11:
I-Tasser predicted ribbon representation of the structure of C-Met protein. Represented in yellow is the extracellular domain (1-932) of the protein fused with a C-terminal polyhistidine-tagged Fc region of human IgG 1. Glycosylation sites are depicted in red.
Figure S12:

International Journal of
Molecular Sciences

MDPI

Comparison of 3D structures depicting an aptamer–protein interaction (A) and antibody–protein interaction (B).

<u>Figure S13:</u>

C-Met 3D structure depicting epitope identification. The epitope peptides for CLN0003 and CLN00004 aptamers are highlighted (Blue and light Blue) in the 3D-structure of C-Met.

Figure S1

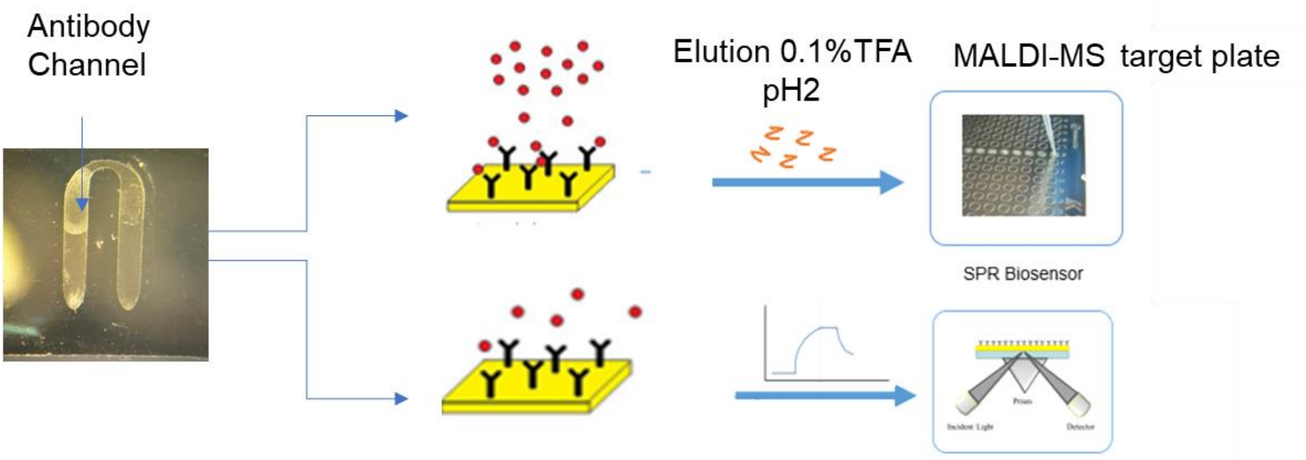

International Journal of
Molecular Sciences

MDPI

Figure S2

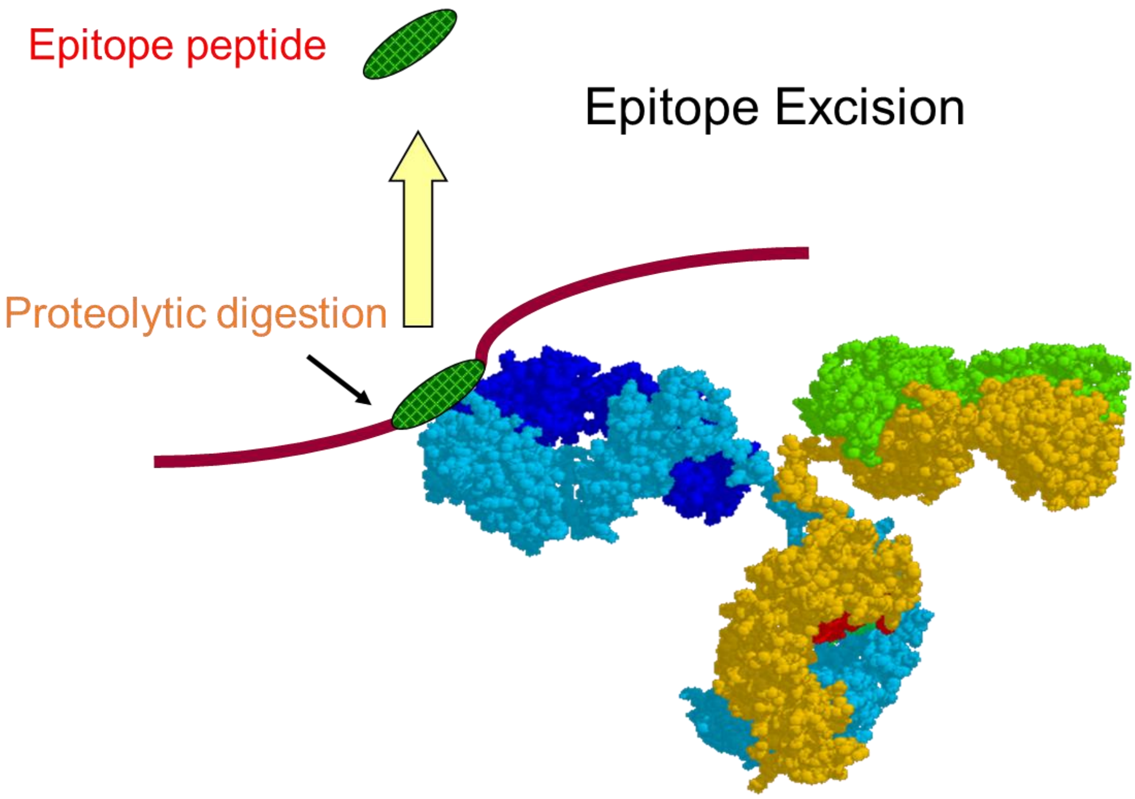

International Journal of Molecular Sciences

MDPI

Figure S3

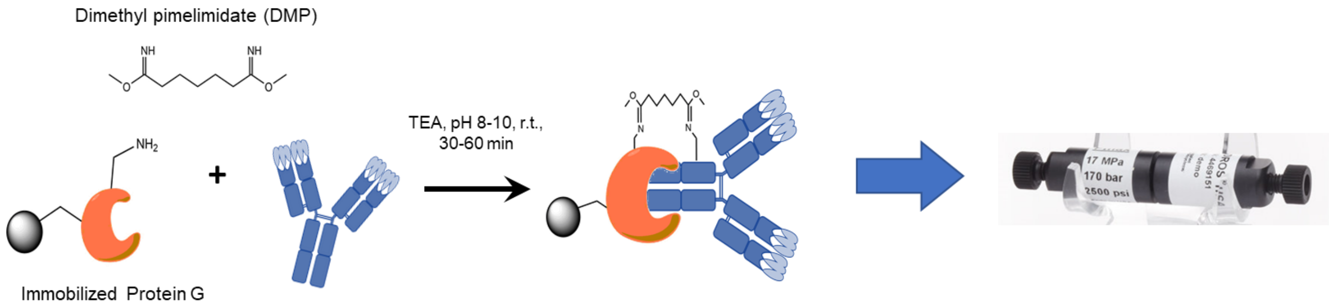

International Journal of Molecular Sciences

Figure S4

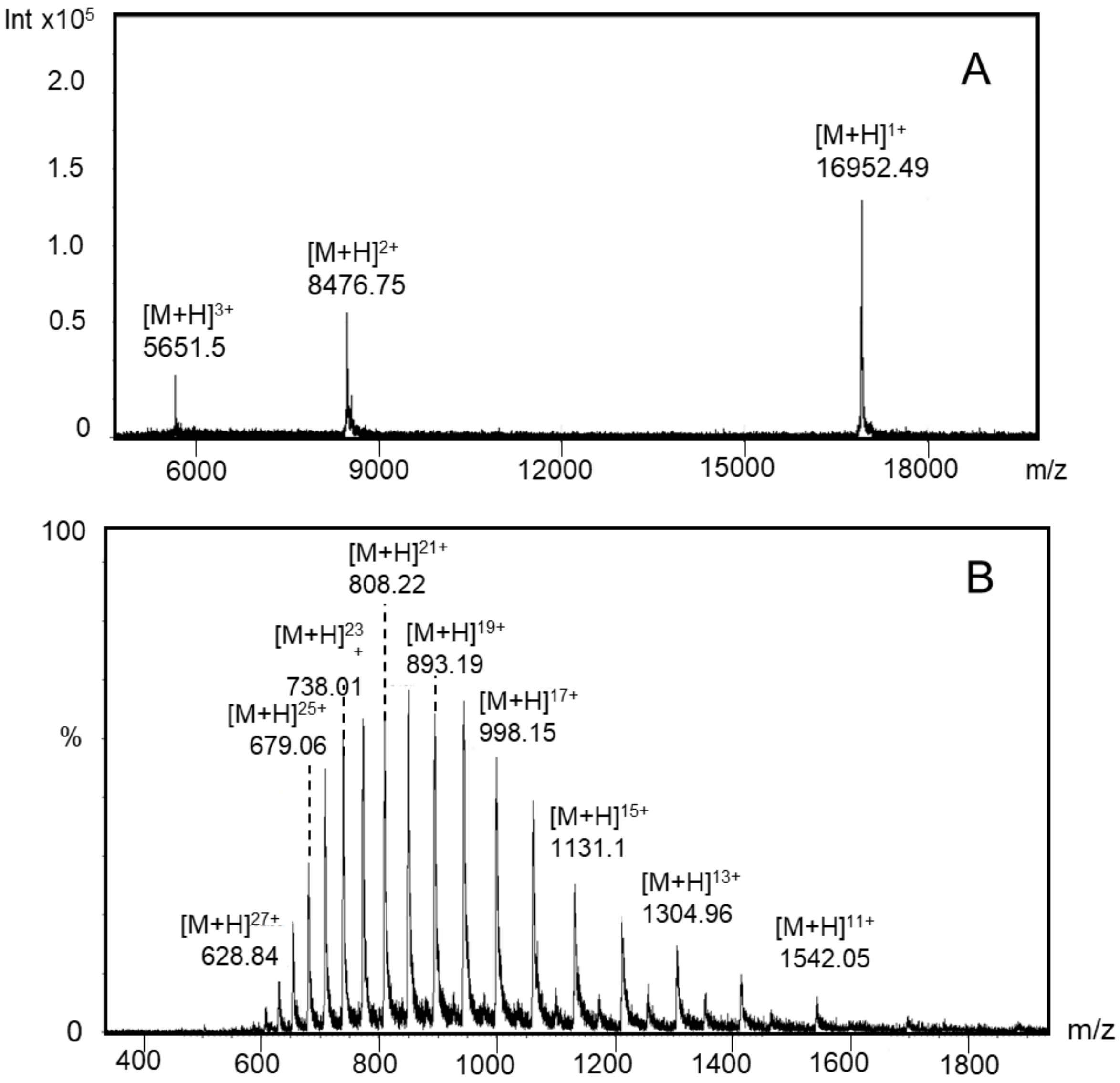

International Journal of *Molecular Sciences*

MDPI

Figure S5

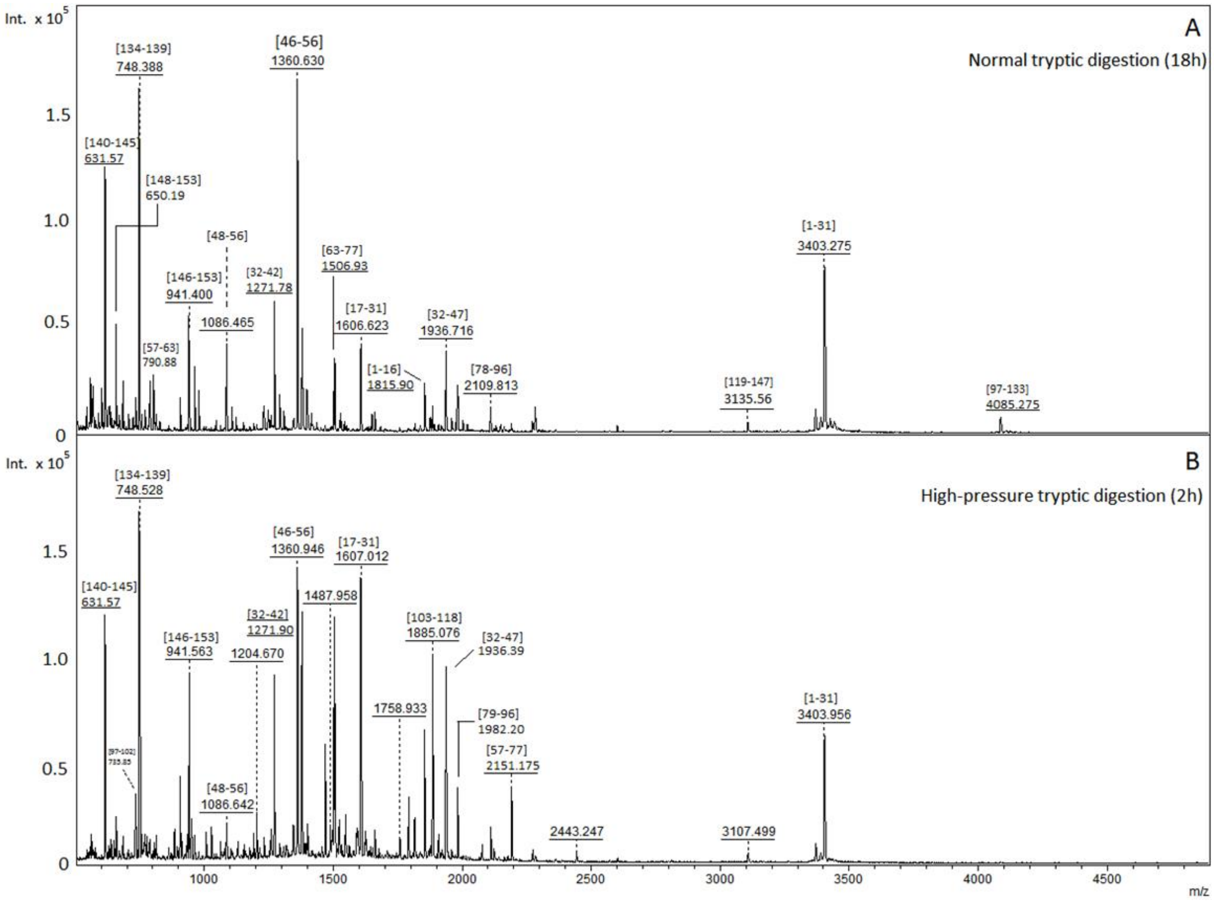

Figure S6

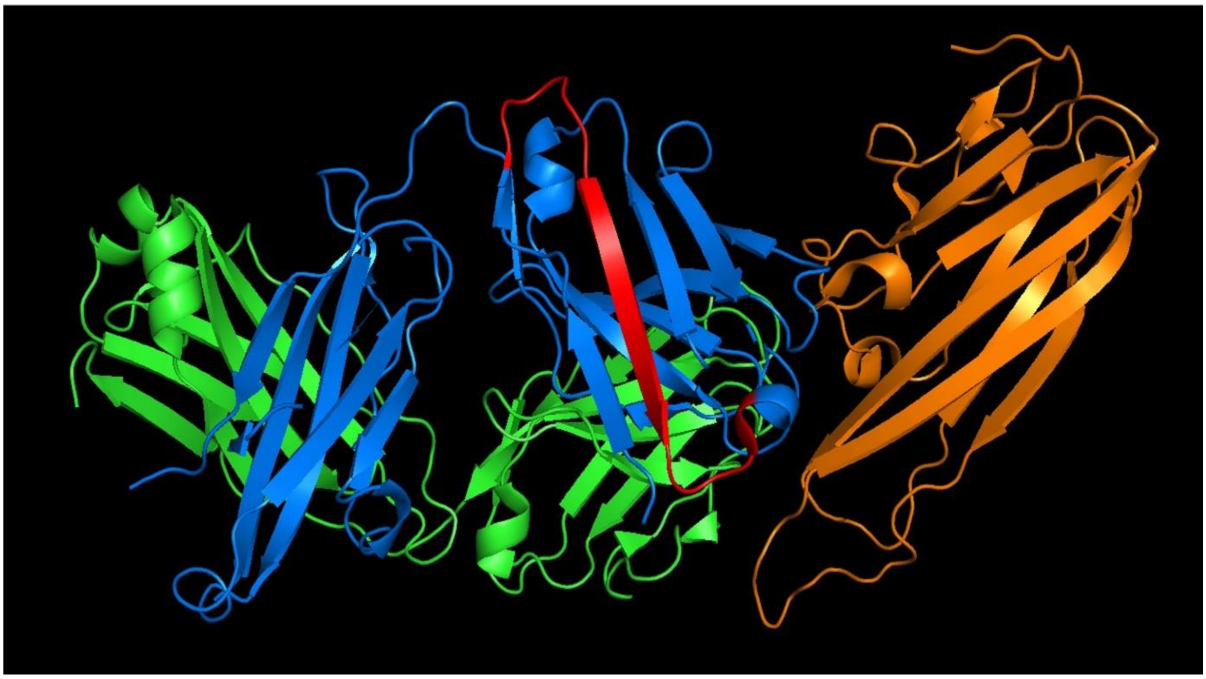

International Journal of *Molecular Sciences*

MDPI

Figure S7

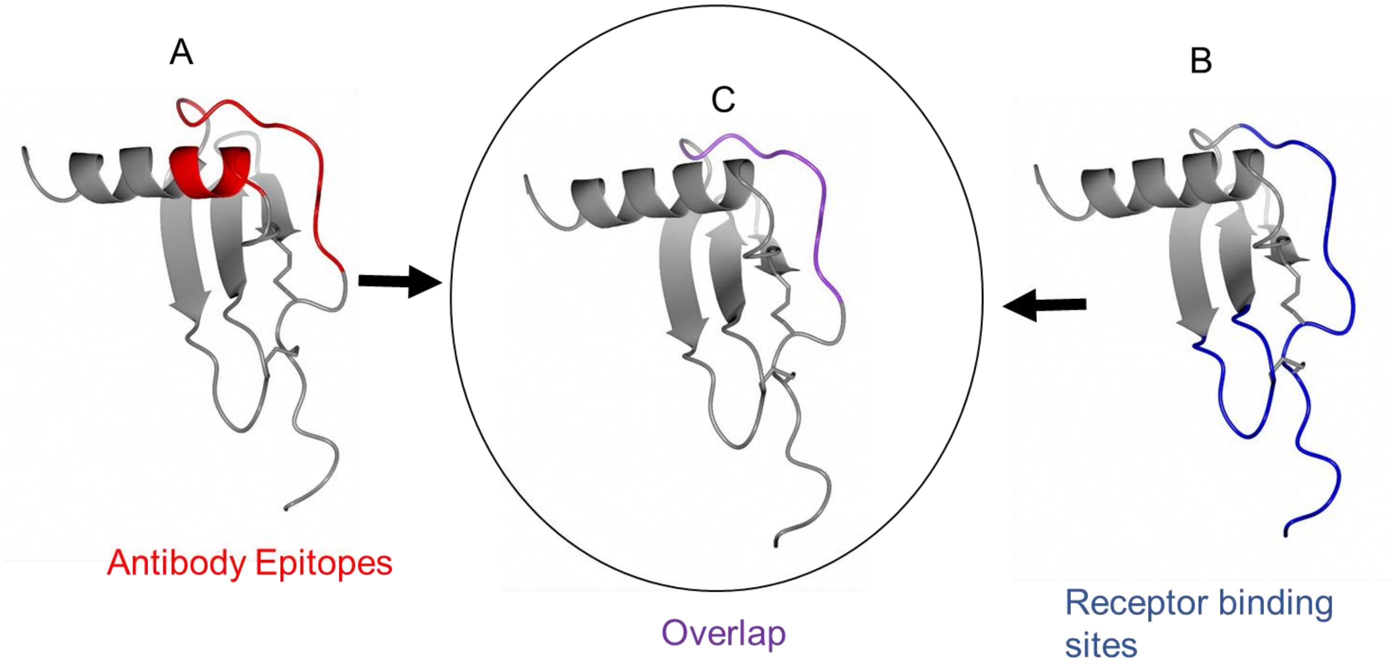

International Journal of
Molecular Sciences

MDPI

Figure S8

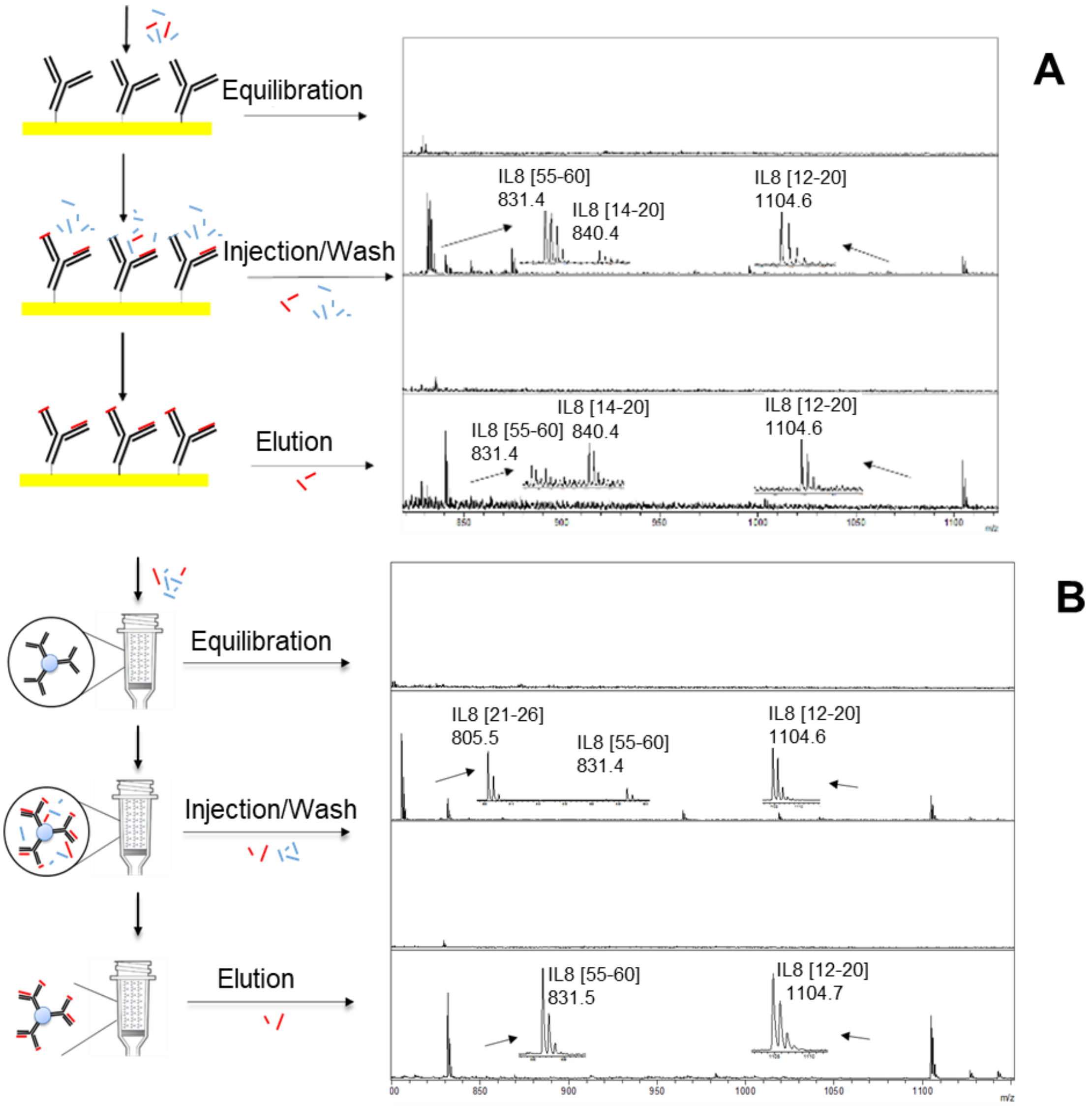

International Journal of *Molecular Sciences*

MDPI

Figure S9

A

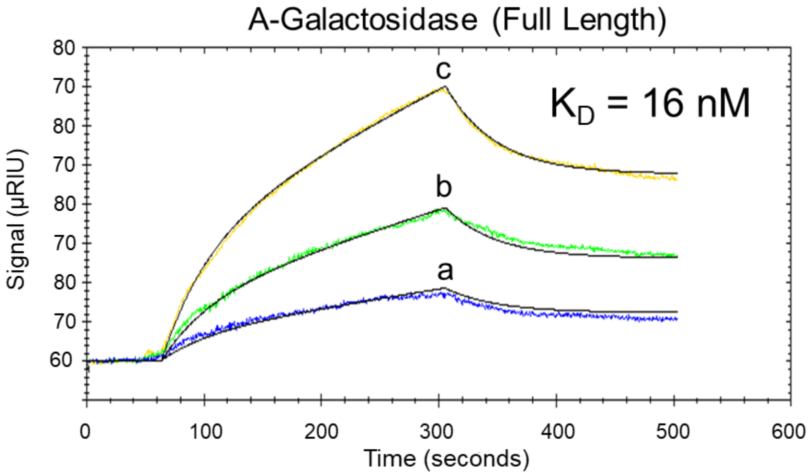

- a = 5 µM
- b = 10 µM
- c = 20 µM

B

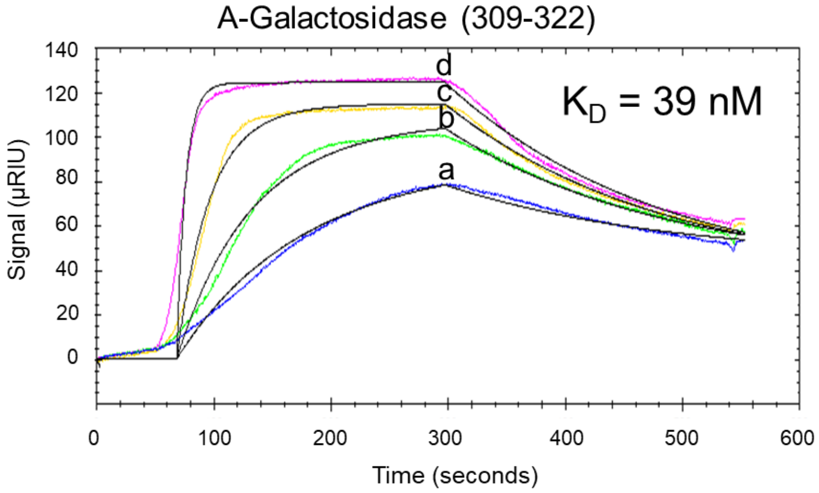

- a = 50 nM
- b = 100 nM
- c = 250 nM
- d = 1 µM

International Journal of
Molecular Sciences
MDPI

Figure S10

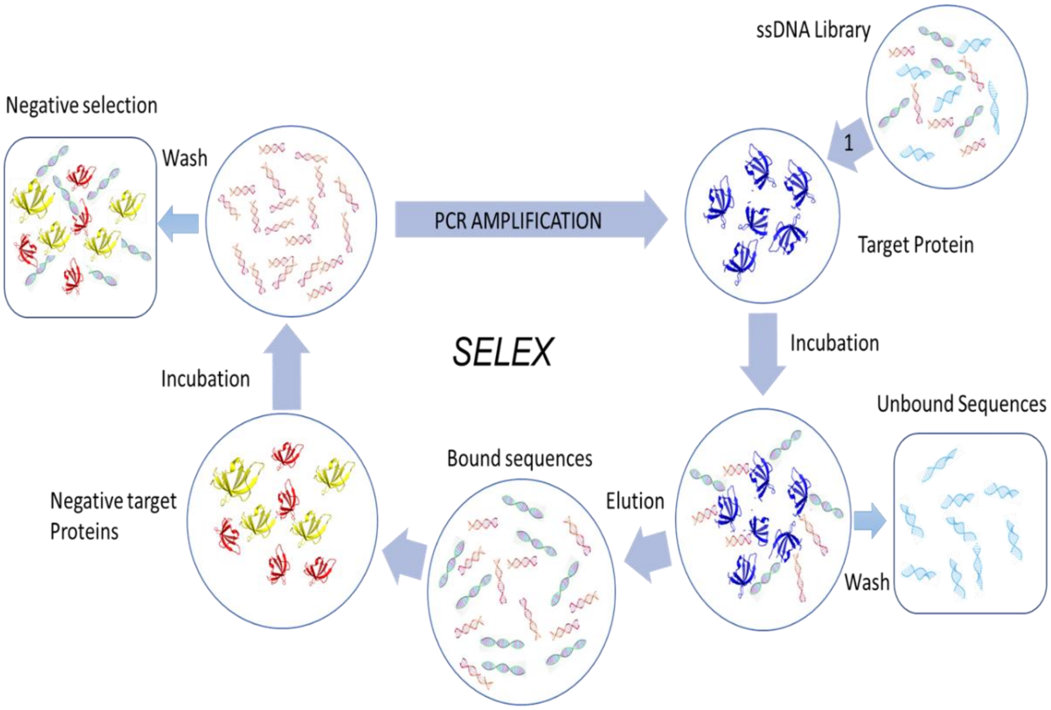

International Journal of Molecular Sciences

Figure S11

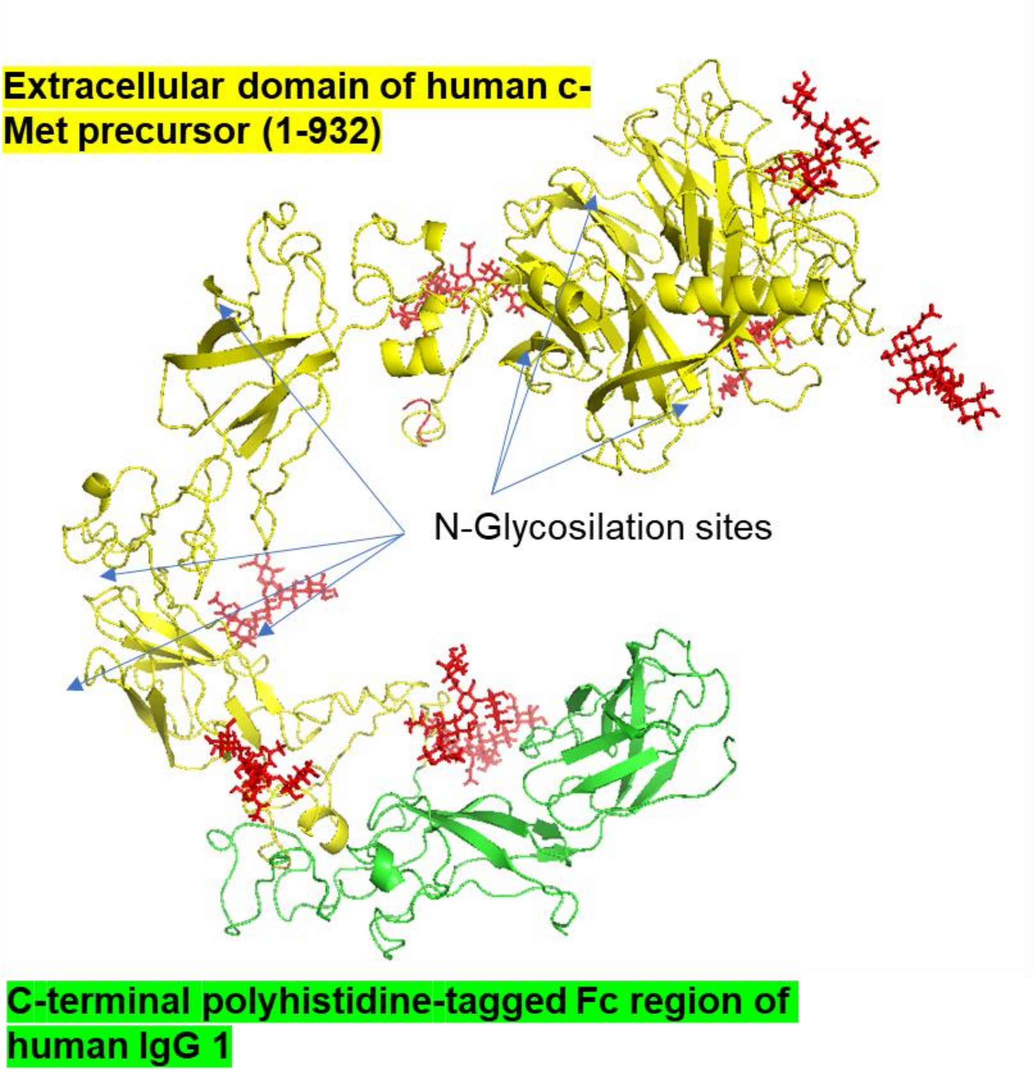

International Journal of *Molecular Sciences*

MDPI

Figure S12

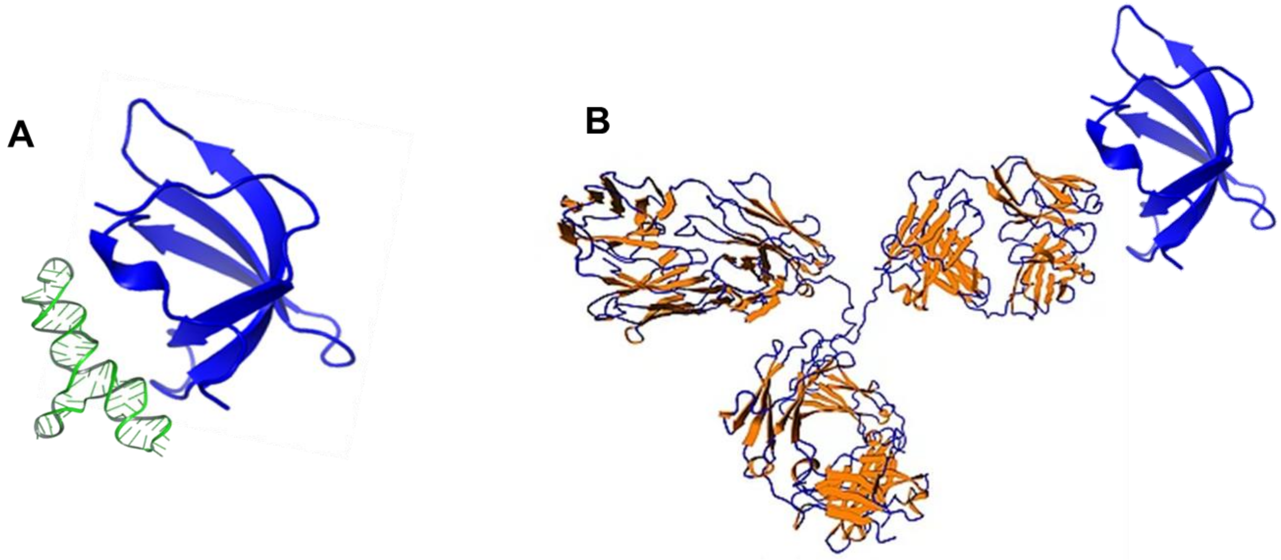

International Journal of *Molecular Sciences*

Figure S13

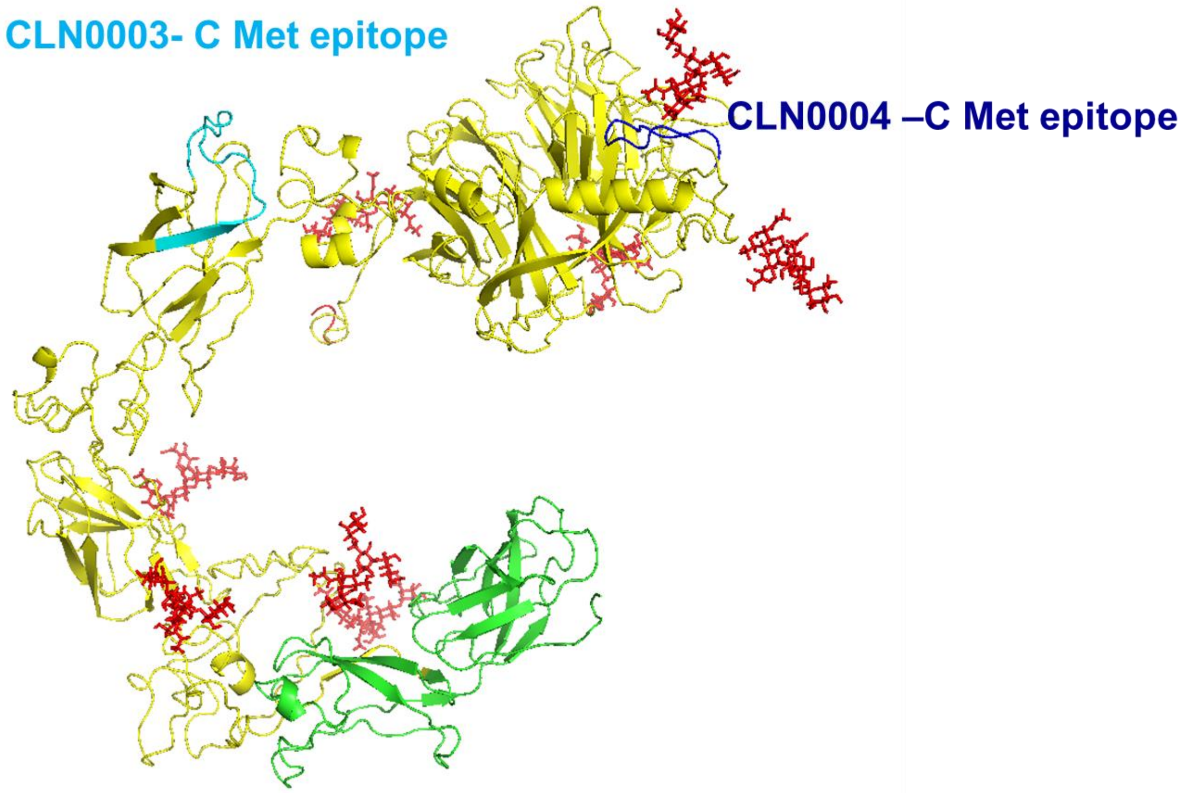

Supplement: Supplementary file 1 [file ijms-22-12832-s001.zip › ijms-1448710-supplementary.pdf]
